# A biomimetic model composed of injectable 3D muscle-like tissue, stromal and immune cells for recapitulating the rapid immune signature predictive of mRNA vaccine immunogenicity

**DOI:** 10.3389/fimmu.2025.1651095

**Published:** 2025-10-10

**Authors:** Marilena Paola Etna, Claudia Fuoco, Martina Severa, Daniela Ricci, Alessandro Sinigaglia, Camilla Lucca, Giada Cairo, Barbara Bottazzi, Cecilia Garlanda, Anna Teresa Palamara, Luisa Barzon, Cesare Gargioli, Eliana Marina Coccia

**Affiliations:** ^1^ Istituto Superiore di Sanità, Department of Infectious Diseases, Rome, Italy; ^2^ Department of Biology, University of Rome Tor Vergata, Rome, Italy; ^3^ Department of Molecular Medicine, University of Padova, Padua, Italy; ^4^ Department of Inflammation and Immunology, Humanitas Clinical and Research Centre – Istituto di Ricovero e Cura a Carattere Scientifico (IRCCS), Milan, Italy; ^5^ Department of Biomedical Sciences, Humanitas University, Milan, Italy

**Keywords:** vaccine, muscle cells, fibroblasts, immune cells, innate immune signature, *in vitro* models

## Abstract

**Background:**

System vaccinology identified an early innate signature associated with vaccine-mediated protection whose induction is likely to involve both immune and non-immune cells.

**Methods:**

To dissect muscle and stromal cell contribution, we simulated *in vitro* anti-COVID19 BNT162b2 mRNA vaccine intramuscular administration in human primary cell systems composed of 3D muscle-like tissue (3D-MT), fibroblasts, and peripheral blood mononuclear cells (PBMC).

**Results:**

BNT162b2 vaccine was recognized by all cell types, although fibroblasts only translated the spike antigen. Factors from vaccine-injected 3D-MT stimulated monocyte and macrophage recruitment and promoted inflammatory macrophage activation, while stromal factors improved dendritic cell frequency and activation. Conditioned media from vaccine-exposed 3D-MT and fibroblasts elicited in PBMC the expression of an early innate immune module previously associated with protective responses in BNT162b2 vaccinees.

**Conclusion:**

Thus, models combining human PBMC, stromal and muscle cells could be employed for the *in vitro* validation of system vaccinology findings and non-animal vaccine pre-clinical testing.

## 1 Introduction

Effective protection following vaccination primarily depends on the vaccine’s ability to elicit a robust and long-lasting adaptive immune response. This includes both antibody (Ab)-dependent protection and cell-mediated responses, both of which are critically dependent on the efficient stimulation of innate immune system.

In the incomparable effort to rapidly develop a safe and effective vaccine for large-scale administration during the recent COVID-19 pandemic, two lipid-nanoparticle (LNP)-encapsulated mRNA-based vaccines encoding the prefusion-stabilized version of severe acute coronavirus 2 (SARS-CoV-2) spike (S) protein were developed and authorized under emergency use by the end of 2020 in Europe and in United States (https://www.ema.europa.eu/en/medicines/human/EPAR/comirnaty#authorisation-details; https://www.federalregister.gov/documents/2021/01/19/2021-01022/authorizations-of-emergency-use-of-two-biological-products-during-the-covid-19-pandemic-availability). These vaccines, namely, BNT162b2 (Pfizer/BioNtech) and mRNA-1273 (Moderna), were extensively studied for their ability to mount potent neutralizing Ab responses and a functional differentiation of antigen (Ag)-specific CD4^+^ and CD8^+^ T cells (1–7). Relatively few studies have explored, *vice versa*, the mechanism underlying the activation of innate immune cells following mRNA vaccine injection (8). The investigation of the interplay between mRNA-LNP-based vaccines and the innate immune system is particularly important in light of the central role of innate immunity not only in priming adaptive responses but also in shaping local and systemic events occurring in the early post-vaccination phase (8). In this regard, a number of clinical studies conducted on intramuscularly administered BNT162b2 and mRNA-1273 vaccines have reported that the most frequent post-vaccination symptom, observed after both first and boosting vaccine doses, include transient local adverse events and pain at the injection site (9–11).

The route of vaccine administration profoundly impacts on both immunogenicity and side effect profiles (12, 13). Muscle tissue, characterized by rich vascularization and minimal/absent fat layer, provides high mobilization and processing of Ags, resulting in higher seroconversion rates compared to subcutaneous injection (12, 13). These data suggest that tissue-resident cells, in addition to immune cells mobilized from the blood, likely contribute to the early immune response.

Skeletal muscle tissue is composed of multinucleated myofibers and a variety of mononuclear cells, including resident immune cells (as mononuclear phagocytes, mainly macrophages), endothelial cells, satellite cells, and fibro-adipogenic progenitors (14–16). Being the main producers of extracellular matrix proteins, fibroblasts also play an important role in skeletal muscle homeostasis, especially upon tissue injury (17, 18). Several evidence supports the concept that a dynamic crosstalk exists between local tissue cells and immune cells – both resident and recruited – and contributes to the fate of immune response with fibroblasts acknowledged as “non-canonical” players of the innate immunity (19). Moreover, muscle cells themselves can exhibit “innate-like” functions in response to pathogens, inflammatory stimuli and growth factors. These functions include the release of cytokines and chemokines as well as the expression of adhesion and co-stimulatory molecules and innate immune receptors such as toll-like receptors (TLR) (20).

Moving from this evidence, we sought to investigate the early immunological events following intramuscular administration of BNT162b2 mRNA vaccine, utilizing different human cell-based and immune-relevant *in vitro* models, that capture both canonical and non-canonical immune elements. Specifically, human primary fibroblasts, myogenic progenitors (MP), myotubes (MT), 3D muscle-like tissue (3D-MT)—being the local tissue cells—and peripheral blood mononuclear cells (PBMC)—representing the immune compartment—were *in vitro* stimulated with the BNT162b2 vaccine. To our knowledge, this is the first study to assess how local tissue responses shape Ag-presenting cell (APC) migration and activation status as well as the modulation of peripheral immune cells’ capacity to release a vaccine-induced early innate immune module, previously shown to correlate with the protective humoral response elicited by BNT162b2 vaccine *in vivo*.

## 2 Materials and methods

### 2.1 Ethics statement

This study involving human participants was reviewed and approved by Istituti Fisioterapici Ospitalieri (IFO) Ethical Committee for the use of muscle samples from healthy donors undergoing surgeries (CE number: 1900/DSa IRE). Muscle biopsies were taken during orthopedical intervention, and all of the participants provided their written informed consent by the IFO in line with the Declaration of Helsinki. Istituto Superiore di Sanità Review Board approved the use of blood from healthy donors (CE number: AOO-ISS – 22/12/2023–0059852 PRE BIO CE 01.00). Written informed consent to participate in this study was provided by the personnel of the Blood Transfusion Service and Hematology Department of Umberto I Hospital (Rome, Italy).

### 2.2 Fibroblast culture and treatment

Human foreskin fibroblasts BJ (ATCC CRL-2522) were cultured in Dulbecco’s modified Eagle’s medium (DMEM, Gibco) supplemented with 10% fetal bovine serum (FBS, Gibco), 1% Glutamax (Gibco), and 1% penicillin/streptomycin (Gibco). Sub-confluent cultures (3 × 10^6^ cells/dish) were obtained in 10-cm dishes (6 × 10^5^ cells/mL), with the medium refreshed 6 hours (h) before treatment with anti-COVID-19 BNT162b2 mRNA vaccine (VAX, Pfizer-Biontech). The fibroblasts were treated with 1 µg/mL of VAX immediately after its reconstitution. Culture supernatants and cell pellets were collected at the indicated times and stored at -80 °C for later analysis as specified below.

### 2.3 Isolation of human myogenic progenitors, differentiation of muscle cell in 2D cultures and treatment

Pericytes (myogenic progenitors, MP) were isolated from muscle biopsies obtained as *res nullius* from healthy donors undergoing orthopedical surgeries at IFO. Briefly, human muscle biopsies were freshly processed (within 24 h from harvesting) by enzymatic digestion with 100 U/mL of Collagenase Type II to obtain a suspension of mononucleated cells and afterward plated at low confluence (1.000 cells/cm^2^) for the selection of alkaline phosphatase (ALP)-positive MP and colony formation. Isolated cells were tested by cytofluorimetric (FACS) analysis to evaluate the expression of specific MP markers, namely, ALP (Novus Biologicals, NB110-3638PE), neural/glial Ag 2, melanoma cell adhesion molecule (CD146) (Novus Biologicals, NBP2-72595AF700), platelet-derived growth factor receptor b (CD140b) (BD Biosciences, 743035BV605), Thy-1 Ag (CD90; Beckman Coulter, IM1839U FITC), and neural cell adhesion molecule (CD56; Beckman Coulter, B92446 AF700) (21). To obtain a homogeneous population of MP (double-positive cells CD90^+^/CD56^+^), isolated cells were cultured into CYTO-GROW (Resnova) medium supplemented with 100 U/mL penicillin and 100 μg/ml streptomycin (Gibco) at 37°C and 5% CO_2_ humid atmosphere as previously described (21, 22). To induce myogenic differentiation, 3 × 10^5^ MP were plated in six-well Petri dishes and cultured for up to 21 days in supplemented CYTO-GROW medium and placed at 37°C and 5% CO_2_ humid atmosphere until reaching the complete differentiation condition (21). MP and MT (1 × 10^6^ cells at the density of 1 × 10^6^ cells/mL) were treated with VAX (1 µg/mL) immediately after its reconstitution. Culture supernatants and cell pellets were collected at the indicated times and stored at -80 °C for later analysis as specified below.

### 2.4 Generation of 3D muscle-like tissue and vaccine injection

All of the experiments were performed using poly-ethylene glycol fibrinogen (PF) biomimetic matrix. In particular, for the generation of each 3D-MT, 8 mg/mL PF, supplemented with 0.1% Irgacure™ 2959 photoinitiator solution (Ciba Specialty Chemicals), was loaded with 1 × 10^6^ MP. The fork was placed in the mold, and the cell–hydrogel mixture was poured into a polytetrafluoroethylene (Teflon) caster (23). The mixture was polymerized by exposing the mold chamber to non-toxic and low-penetrating UV light for 5 min (**Supplementary Figures S1A, B**). Polymerized constructs were gently removed and transferred to normal 35-mm dishes containing 1 mL of CYTO-GROW medium supplemented as previously described. The vaccine was used fresh after reconstitution at the final concentration of 1 µg/mL. Then, 3D-MT were vaccine-injected with 100 µL of VAX dilution through an insulin syringe (**Supplementary Figure S1C**). Culture media and the corresponding 3D-MT were collected at the indicated time points and stored at -80 °C for later analysis as specified below.

### 2.5 Isolation of PBMC and treatment

PBMC were isolated from freshly collected buffy coats derived from blood donation of healthy volunteers at Blood Transfusion Service and Hematology Department of Umberto I Hospital (Rome, Italy) by density gradient centrifugation using lympholyte-H (Cedarlane) as previously described (24). Briefly, PBMC were cultured at 2 × 10^6^ cells/mL in RPMI 1640 supplemented with penicillin/streptomycin (100 U/mL), L-glutamine (2 mM), and 10% FBS for 24 or 72 h and then stimulated with 1 µg/mL of freshly reconstituted VAX or with conditioned media (CM) from fibroblast cultures or muscle cell models used at v/v ratio 1:5 and 1:10. For PBMC treatment, CM were collected at 24 or 72 h from unstimulated or VAX-treated fibroblasts, while CM were harvested at 24 h from not stimulated (NS) and VAX-exposed 2D MT and 3D-MT. To evaluate also a possible contribution of the residual VAX contained in CM after thawing, frozen VAX (FRZ. VAX) was also used at 1, 0.2, and 0.1 µg/mL to treat PBMC. After 24 h, culture supernatants from unstimulated and treated PBMC were collected and stored at -80 °C for later analysis, while cell pellets were employed for gene expression studies, confocal microscopy analysis, or evaluation of APC phenotype (see below).

### 2.6 Spike expression in fibroblasts, muscle cell models, and PBMC

Human BJ fibroblasts were cultured in 24-well plates attached on round glass coverslips (50.000 cells/well) and treated with VAX for 24 h as described above or NS as negative control. PBMC, NS or stimulated with VAX for 24 h as previously indicated, were collected, and 1 × 10^5^ cells were adhered on microscopy slides by cytospin. Both cell types were fixed in 4% paraformaldehyde (PFA, Sigma Aldrich) at room temperature (RT) and then permeabilized with 0.1% Triton X-100 (BioRad) and blocked with 4% bovine serum albumin (BSA, Sigma Aldrich) overnight (o.n.) at 4 °C or 1 h at RT. Immunofluorescence assay was performed by incubation with primary Ab against SARS-CoV-2 S1 protein (40150-R007, Sino Biological) diluted 1:500 followed by secondary anti-rabbit IgG conjugated with AlexaFluor 488 (Invitrogen, Thermo Fisher Scientific A11008) diluted 1:1,000. Finally, Draq5 fluorescent probe (Invitrogen, Thermo Fisher Scientific) diluted 1:2,000 was added for 10 min at RT for staining of cell nuclei. Immunofluorescence preparations were observed with Nikon Eclipse Ti2 confocal microscope, and images were acquired and analyzed with LAS V3.8 (Leica) software.

Not stimulated or VAX-exposed 2D MP and MT as well as 3D-MT were instead fixed with 2% PFA and processed for immunofluorescence analysis. Briefly, the samples were permeabilized with 0.3% Triton X-100 (Sigma Aldrich) in PBS 1X without Ca^2+^ and Mg^2+^ (Biowest) for 1 h at RT and then incubated with a blocking solution, consisting of PBS 1X, 0.1% Triton X-100, 1% glycine, and 10% natural goat serum for 1 h at RT. Afterward, 2D MP were incubated for 1 h at RT with Phalloidin Alexa Fluor 555 (Invitrogen, Thermo Fisher Scientific, A12379) for labeling F-actin of cytoskeleton, while MT and 3D-MT were incubated for 1 h and o.n. at RT, respectively, with anti-myosin heavy chain (anti-MF20) mouse primary antibody (DSHB, AB_2147781) diluted 1:200 to assess terminal myogenic differentiation. For the detection of SARS-Cov-2 S1 protein, Ab was used as previously specified by staining MP and MT for 1 h, while 3D-MT were incubated o.n. at RT.

The samples were then washed three times and incubated 1 h at RT with Alexa-fluor555-conjugated anti-mouse IgG Ab (Invitrogen, Thermo Fisher Scientific A21422) secondary antibody at 1:200 dilution for anti-MF20 detection and with AlexaFluor 488-conjugated anti-rabbit IgG Ab (Invitrogen, Thermo Fisher Scientific, A11008) diluted 1:1,000 for S1 protein detection. After the indicated time, the washing step was repeated as previously indicated, and labeling of nuclei was performed by incubating the samples with 0.1 μg/mL of DAPI (4′,6-diamidino-2-phenylindole dihydrochloride) for 15 min at RT. Finally, the 3D-MT samples were mounted with Aqua Poly/Mount (Polysciences, Inc). Samples were photographed using a DMI6000B fluorescent microscope (Leica), and images were acquired and analyzed with Leica Application Suite X software.

### 2.7 Analysis of soluble factors released in culture supernatants

The release of soluble factors was studied in culture supernatants from 24- and 48-h vaccine-stimulated fibroblasts, MP, MT, and 3D-MT as well as in PBMC exposed for 24 h, as described above.

Secretions of IL-8 and RANTES/CCL5 were measured by ELISA using DuoSet ELISA Kit (R&D Systems), while the release of IL-6, TNF-α, MCP-1/CCL2, MIP-1β/CCL4, IP-10/CXCL10, IFNγ, and IL-15 was quantified by custom plate simple plex cartridge kit on an ELLA automated immunoassay system (Bio-Techne, San Jose, CA, USA) according to the manufacturer’s instructions and analyzed by the Simple Plex Explorer Program software (Bio-Techne) as previously described (25).

Interferon (IFN)-αs production was determined in culture supernatants by Verikine Human IFN-α multi-subtype ELISA kit (PBL Assay Science). PTX3 levels were measured, as previously described (26), using a sandwich ELISA developed in-house. The assay has a lower limit of detection of 0.1 ng/mL and inter-assay variability between 8% and 10%, and it does not cross-react with C-reactive protein.

### 2.8 RNA extraction and gene expression analysis

Total RNA was isolated by Trizol Reagent (Invitrogen, Thermo Fischer Scientific) from the different cell models, quantified using a NanoDrop ONEc spectrophotometer and quality-assessed with an established cutoff of ~1.8 for 260/280 absorbance ratio. Reverse-transcription was conducted by Vilo reverse transcriptase kit (Invitrogen, Thermo Fischer Scientific).

The expression of genes encoding MX dynamin-like GTPase 1 (MX1), IFN regulatory factor 1 (IRF1), IFNB1, and IFNL1 was measured at 24 and 48 h by quantitative real-time PCR (q-PCR) using the appropriate TaqMan assay and TaqMan Universal Master Mix II (Applied Biosystems, Thermo Fisher Scientific) on a ViiA7 Instrument (Applied Biosystems, Thermo Fisher Scientific). RNA sensors were studied at 24 h when the expression of endosomal and cytoplasmic receptors can be observed in all of the analyzed cellular systems following specific stimulation. In particular, for TLR3, TLR7, TLR8, RIG-I, and MDA5 gene expression, LightCycler Fast Start DNA SYBR Green I Master Mix on a LightCycler 2.0 Instrument (Roche Diagnostics, Basel, Switzerland) was employed with the following primer pair sequences:

TLR3
Forward: CCTGGTTTGTTAATTGGATTAACGA
Reverse: TGAGGTGGAGTGTTGCAAAGG
TLR7
Forward: TTACCTGGATGGAAACCAGCTACT
Reverse: TCAAGGCTGAGAAGCTGTAAGCTA
TLR8
Forward: CATCATCGACAACCTCATGC
Reverse: CTGTAACACTGGCTCCAGCA
RIG-I
Forward: TGCAAGCTGTGTGCTTCTCT
Reverse: CGCTAATCCGTGATTCCACT
MDA-5
Forward: CAGAAGGAAGTGTCAGCTGCTTAG
Reverse: TGCTGCCACATTCTCTTCATCT

The housekeeping gene TATA-box-binding protein (TBP) was used as normalizer. Real-time reactions were run at least in duplicates. Sample values for each mRNA were normalized to the selected housekeeping gene using the formula 2^−ΔCt^.

### 2.9 Migration assay

Migration assay was performed by plating 2 × 10^5^ PBMC on the top chamber of 5-µm-pore polycarbonate filters of a 24-well transwell chamber (Corning Inc.). In the lower chamber, 1:5 and 1:10 v/v dilutions of CM from NS and VAX-exposed fibroblasts or 3D-MT were placed. PBMC were then incubated for 4 h at 37 °C and 5% CO_2_ humid atmosphere before the trans-migration assay. After the indicated time, the transwell inserts were removed and the migrated cells counted with a Countess 3 FL Automated Cell Counter (Invitrogen, ThermoFisher Scientific) following standardized protocols. The relative level of migrated cells was calculated on the number of PBMC not exposed to CM and basally migrated during the incubation time. Moreover, by flow cytometric analysis, the relative level of migrated CD14^+^ monocytes, CD11b^+^CD14^-^ macrophages, and CD11c^+^ myeloid dendritic cells (mDC) was assessed.

### 2.10 Flow cytometry analysis

PBMC were stained with a well-established antibody cocktail to study by flow cytometry monocyte, macrophage, and mDC subsets as well as their activation status.

IgG1 or IgG2a isotype controls and monoclonal Abs (mAbs) for HLA-DR (APC-H7, #641411), CD14 (APC, #555399), CD16 (PE, #561313), CD11b (BV750, #747210), CD141 (BDCA3, BV510, #563298), CXCR-3 (PE, #557185), CXCR-4 (PE, #555974), and CD62-L (FITC, #555543) were purchased from BD Biosciences. CD11c (PeVio770, #130-113-581), CD1c (BDCA1, FITC, #130-113-301), and CCR7 (PeVio770, #130-117-396) were purchased from MACS from Miltenyi Biotech and CD86 (PE-CF594, #120-869-73) from eBioscience. To exclude dead cells from the flow cytometry analyses, Fixable Viability Dye (FvDye, eFluor450, #65-0863-14, eBioscience) was always included in the Ab cocktails. The gating strategy used is summarized in **Supplementary Figure S2**. PBMC (1 × 10^6^ cells) were incubated with mAbs at 4°C for 30 min and then fixed with 2% PFA before analysis on a Cytoflex LX cytometer (Beckman Coulter). The data were analyzed by Cytexpert software v.2.1 (Beckman Coulter). The expression of analyzed cell surface molecules was evaluated using the median fluorescence intensity (MFI). Only viable and single cells were considered for further analysis.

### 2.11 Statistical analysis

Statistical analysis was performed using one-way repeated-measures ANOVA when three or more stimulation conditions were compared. In case of significant ANOVA, the pairwise comparisons were carried out using *post-hoc* approaches for multiple comparisons to test the significance of the difference between two stimulation effects. The results were shown as median values ± interquartile range (IQR) or, where indicated, as mean values ± standard error of the mean (SEM). A *p*-value ≤0.05 was considered statistically significant. In the figures, star scale was assigned as follows: *p ≤ 0.05; **p ≤ 0.01; ***p ≤ 0.001; ****p ≤ 0.0001. Data and statistical analyses were processed by Prism software version 9.4.1 (Graph Pad).

## 3 Results

### 3.1 Fibroblasts and muscle cells differentially respond to BNT162b2 vaccine stimulation in terms of spike protein expression, RNA sensor modulation, and innate immune mediator release

**Figure 1:**
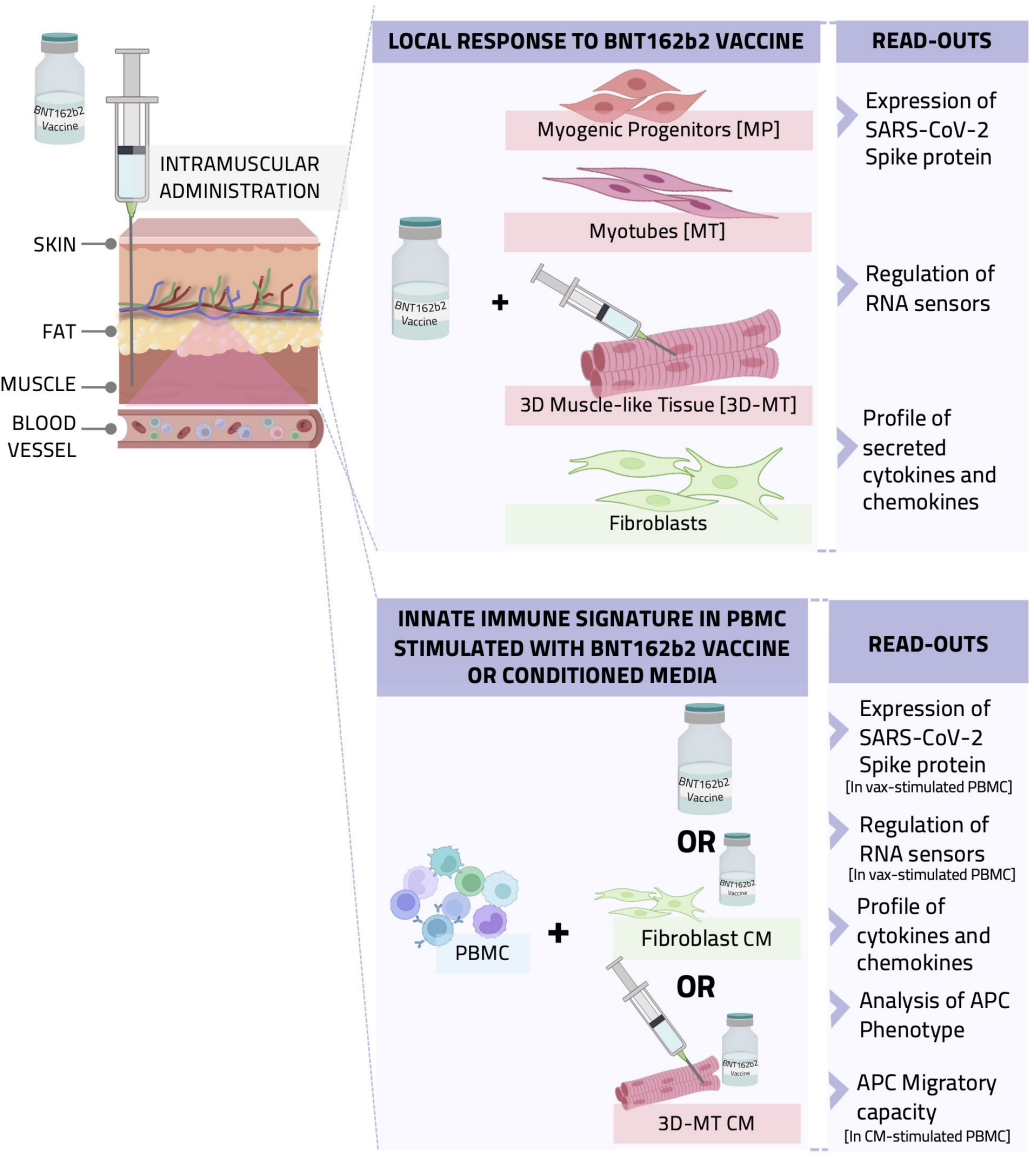
Graphical representation of the *in vitro* study conducted with cell-based models recapitulating the intramuscular environment encountered by BNT162b2 vaccine. A schematic representation of the experimental setting, procedure, and data analysis is shown. BNTb162b2 vaccine (VAX) was used to treat human primary fibroblast cultures and different muscle cell models, namely, myogenic progenitors (MP) and myotubes (MT) as well as 3D muscle-like tissue (3D-MT). The expression of SARS-CoV-2 spike (S) protein and RNA sensors and the production of cytokines and chemokines were analyzed (upper panel). In addition, peripheral blood mononuclear cells (PBMC) were stimulated with VAX or incubated with conditioned media (CM) from unstimulated or vaccine-treated fibroblasts and 3D-MT to assess their impact on innate immune signature. Antigen-presenting cell (APC) immunophenotype and migratory capacity as well as cytokine and chemokine profile were studied (lower panel).

**Figure 2:**
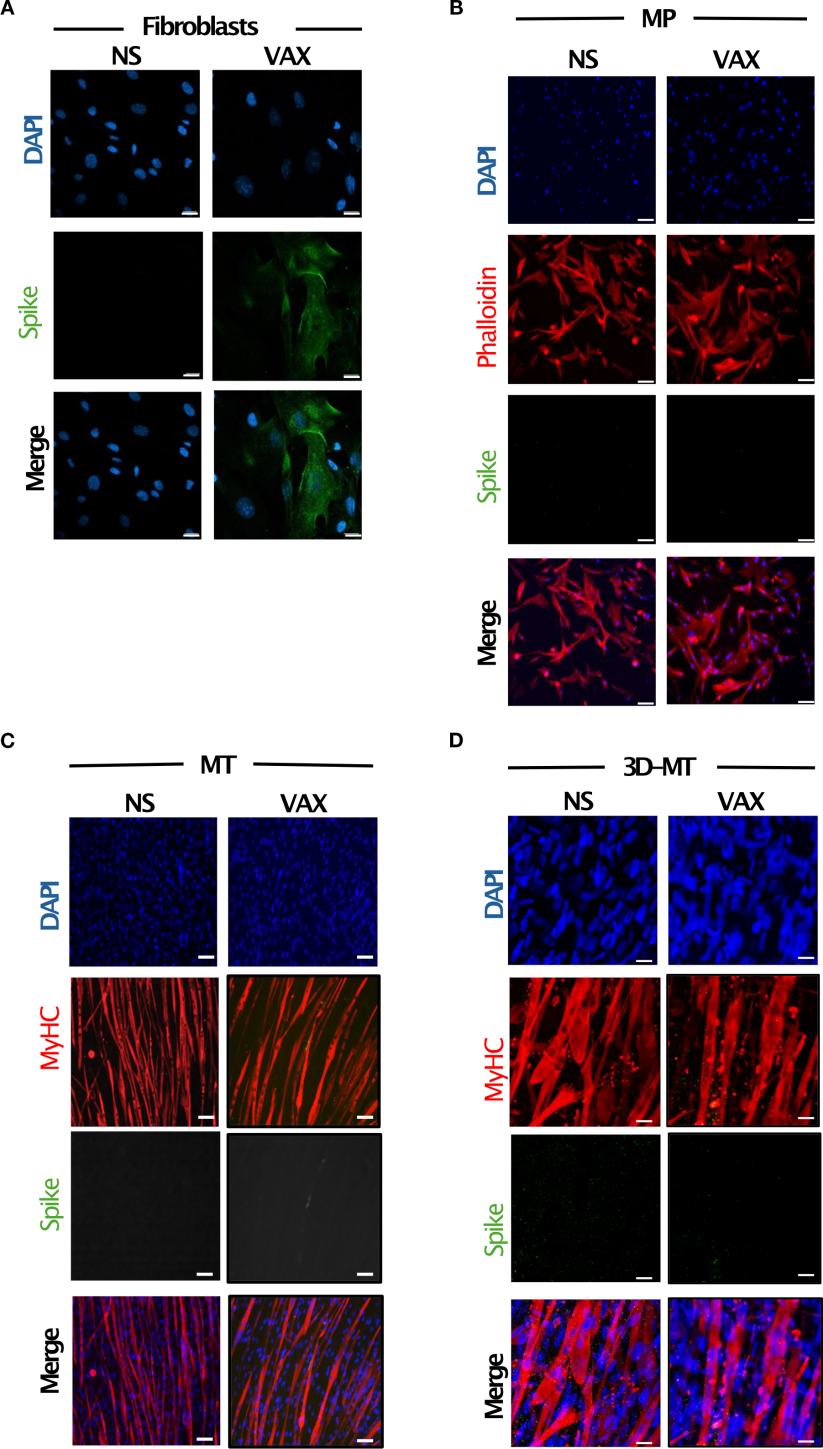
Expression of SARS-CoV-2 spike (S) protein in fibroblasts and muscle models *in vitro* stimulated with BNT162b2 vaccine. Fibroblasts **(A)**, myogenic progenitors (MP) **(B)**, myotubes (MT) **(C)** and 3D muscle-like tissue (3D-MT) **(D)** were left not stimulated (NS) or treated with BNT162b2 vaccine (VAX, 1 µg/mL) for 24 h **(H)**. Immunofluorescence analysis was conducted by confocal microscopy to assess the expression of S protein. Fibroblasts were stained with DAPI (blue) to identify nuclei and with an anti-S antibody (Ab) (green) **(A)**. For MP staining, in addition to DAPI (blue) and anti-S Ab (green), an anti-phalloidin Ab (red) was used as a marker of undifferentiated muscle cells **(B)**, while for MT and 3D-MT, staining with anti-myosin heavy chain (MyHC) Ab (red) was employed as a differentiation marker. Representative images, out of three experiments (*n* = 3) separately performed, are shown in panels **(A–D)**. Scale bar: 20 μm.

Assuming that tissue-resident non-immune cells—such as fibroblasts and muscle cells—are among the first cells to encounter *in vivo* an intramuscularly administered vaccine, like the BNT162b2 vaccine, we sought to *in vitro* reproduce and characterize the local response at the injection site by using different human-cell-based models. Specifically, we employed human MP, MT, and primary fibroblasts as well as 3D-MT models that more closely mimic the *in vivo* structure and cell composition present in the muscle tissue (**Figure 1**). We assessed the expression of the BNT162b2-vaccine-encoded Ag, namely, the SARS-CoV-2 S protein, by immunofluorescence analysis at 24 h post-vaccine treatment. Among the analyzed cell models, human primary fibroblasts only showed detectable S protein expression (**Figure 2A**). Indeed no signal was detected in vaccine-stimulated MP and MT as well as on 3D-MT (**Figures 2B–D**).

**Figure 3:**
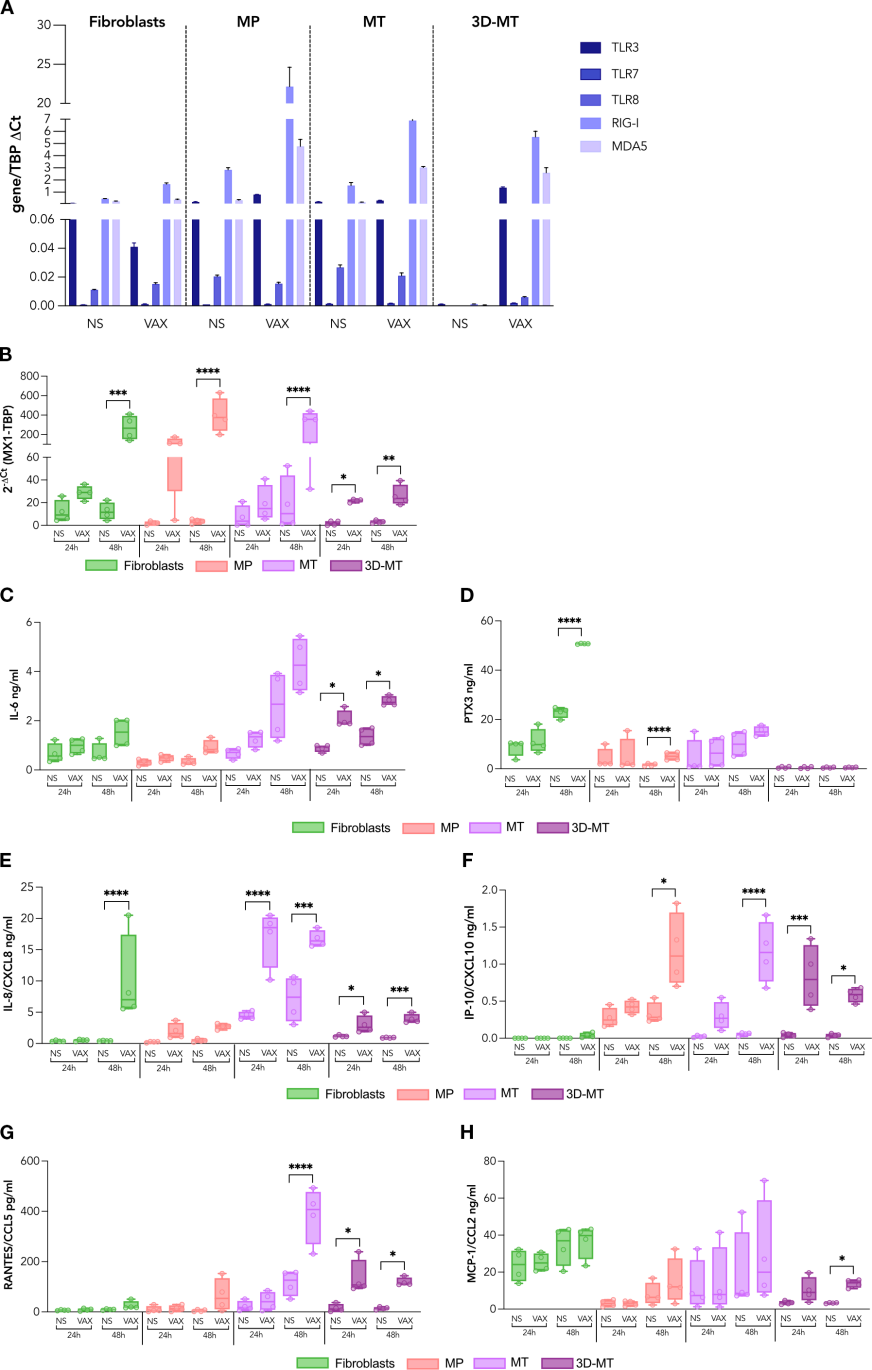
Profile of RNA sensors and innate immune mediators induced by BNT162b2 vaccine in fibroblasts and muscle models. Fibroblasts, myogenic progenitors (MP), myotubes (MT), and 3D muscle-like tissue (3D-MT) were not stimulated (NS) or treated with BNT162b2 vaccine (VAX, 1 µg/mL). The expression of the RNA sensors, namely, TLR3, TLR7, TLR8, RIG-I, and MDA5, was analyzed after 24 h **(H)** of treatment by quantitative RT-PCR **(A)**. The results are means ± SEM of three independent experiments (*n* = 3). MX1 mRNA **(B)** expression was determined at 24 and 48 h by quantitative RT-PCR. The levels of IL-6 **(C)**, PTX3 **(D)**, IL-8/CXCL8 **(E)**, IP-10/CXCL10 **(F)**, RANTES/CCL5 **(G)**, and MCP-1/CCL2 **(H)** were measured in culture supernatants collected from fibroblasts, MP, MC, and 3D-MT not stimulated (NS) or stimulated with VAX for 24 and 48h. The results are shown as median values ± interquartile range of four independent experiments (*n* = 4). Non-parametric one-way ANOVA with Tukey’s adjustment for multiple comparisons was used to calculate statistical significance of differences. The star scale was assigned as follows: *p ≤ 0.05, **p ≤ 0.01, ***p ≤ 0.001, ****p ≤ 0.001.

To assess whether, irrespective of S expression, the tissue-resident non-immune cells are able to respond to the mRNA-based vaccine, the basal and vaccine-induced expression of both endosomal and cytoplasmic RNA sensors, namely, TLR3 (transmembrane receptor localized on endosomes that senses double-stranded RNA), TLR7 and TLR8 (endosomal receptors, recognizing single-stranded RNA), RIG-I (involved in cytoplasmic recognition of both single and double-stranded RNA), and MDA5 (cytoplasmic sensor detecting double-stranded RNA) as well as the secretion of immunoregulatory mediators was investigated (**Figure 3**). With the exception of TLR7 poorly detectable under basal and vaccine-stimulated conditions, all the other analyzed molecules resulted to be expressed, although at a different extent, at the steady-state level in fibroblasts, MP, and MT (**Figure 3A**). While no variation in RNA sensors’ expression occurred in fibroblasts, both RIG-I and MDA5 expression increased in MP, MT, and 3D-MT in a vaccine-dependent manner. In the latter cell model, BNT162b2 vaccine injection also induced the endosomal receptor TLR3 (**Figure 3A**). Being the intracellular sensors involved in nucleic acid recognition mainly expressed in immune cells, PBMC were used as control. PBMC showed at least a 10-fold induction of all the analyzed sensors at 24 h post-vaccine treatment (**Supplementary Figure S3A**). However, these inductions occurred in the absence of S Ag expression as analyzed by confocal microscopy (**Supplementary Figure S3B**). We then investigated the responsiveness of fibroblasts and muscle cell models in terms of cytokine and chemokine production. As expected, due to nucleoside modifications in the mRNA molecules, IFN-αs production was faint and observed in fibroblasts only (**Supplementary Figure S4A**). Nevertheless, the IFN-inducible gene MX1 was induced in muscle cells (**Figure 3B**), suggesting the involvement of other IFNs, such as IFN-β and IFN-λ. Indeed the vaccine-dependent expression of IFNB1 accounts for MX1 upregulation at 24 and 48 h in 3D-MT, while in MP and MT, the MX1 levels appeared to be likely regulated by the combined action of IFNB1 and IFNL1 (**Figure 3B**, **Supplementary Figures S4B, C**).

Then, to study innate-like responses, a panel of secreted pro-inflammatory and chemotactic mediators was analyzed post-vaccine stimulation. In all 2D cultures, most changes occurred at 48 h, whereas in 3D-MT, modulations were detectable as early as 24 h (**Figures 3C–H**). In particular, IL-6, an inflammatory and pyrogenic cytokine involved in the acute-phase protein response, was slightly upregulated in fibroblasts, MP and MT, but significantly induced in 3D-MT at both time points (**Figure 3C**).

PTX3, a soluble receptor participating in peripheral immunity and inflammation, was significantly released by fibroblasts and MP 48 h post-BNT162b2 vaccine treatment, while it resulted poorly or was not stimulated in MT and 3D-MT (**Figure 3D**).

The profiling of IL-8/CXCL8—a neutrophil chemotactic factor and one of the major mediators of inflammatory response, IP-10/CXCL10—an IFN-inducible chemokine involved in the inflammation and recruitment of APC, NK, and T cells, RANTES/CCL5—a potent chemoattractant for leukocytes including monocytes, and MCP-1/CCL2—a monocyte/macrophage chemotactic factor was also performed (**Figures 3E–H**). The data showed a cell-type-peculiar chemokine pattern whose level very often reached significant production following 48 h of vaccine stimulation. Specifically, fibroblasts constitutively secreted MCP-1/CCL2 and released IL-8/CXCL8 in a vaccine-dependent manner (**Figures 3E, H**); MP were a major producer of IP-10/CXCL10 and also contributed to the overall vaccine-induced MCP-1/CCL2 (**Figures 3F, H**); MT secreted high basal levels of MCP-1/CCL2 and released IL-8/CXCL8, IP-10/CXCL10, and RANTES/CCL5 upon vaccine stimulation (**Figures 3E–G**); the 3D-MT model showed a robust induction of all analyzed chemokines at 24 and 48 h post-vaccine injection, with MCP-1/CCL2 reaching significance at 48 h only (**Figures 3E–H**). It is noteworthy that the early upregulation of IFN-inducible chemokines IP-10 and RANTES mirrored the increase in MX1 expression observed in vaccine-injected 3D-MT at 24 h (**Figures 3E, F, H**).

Overall, these data indicate that the immune factors produced by 3D-MT upon vaccine exposure are likely to play a major role in shaping the local immune milieu post-vaccination. Nevertheless, collaborative interactions among the other tissue-resident non-immune cells contribute to sense the mRNA-based vaccine and to initiate a rapid recruitment and activation of immune cells at the injection site.

### 3.2 Antigen-presenting cells migrate in response to chemokines released from BNT162b2-vaccine-stimulated 3D muscle-like tissue and fibroblasts

**Figure 4:**
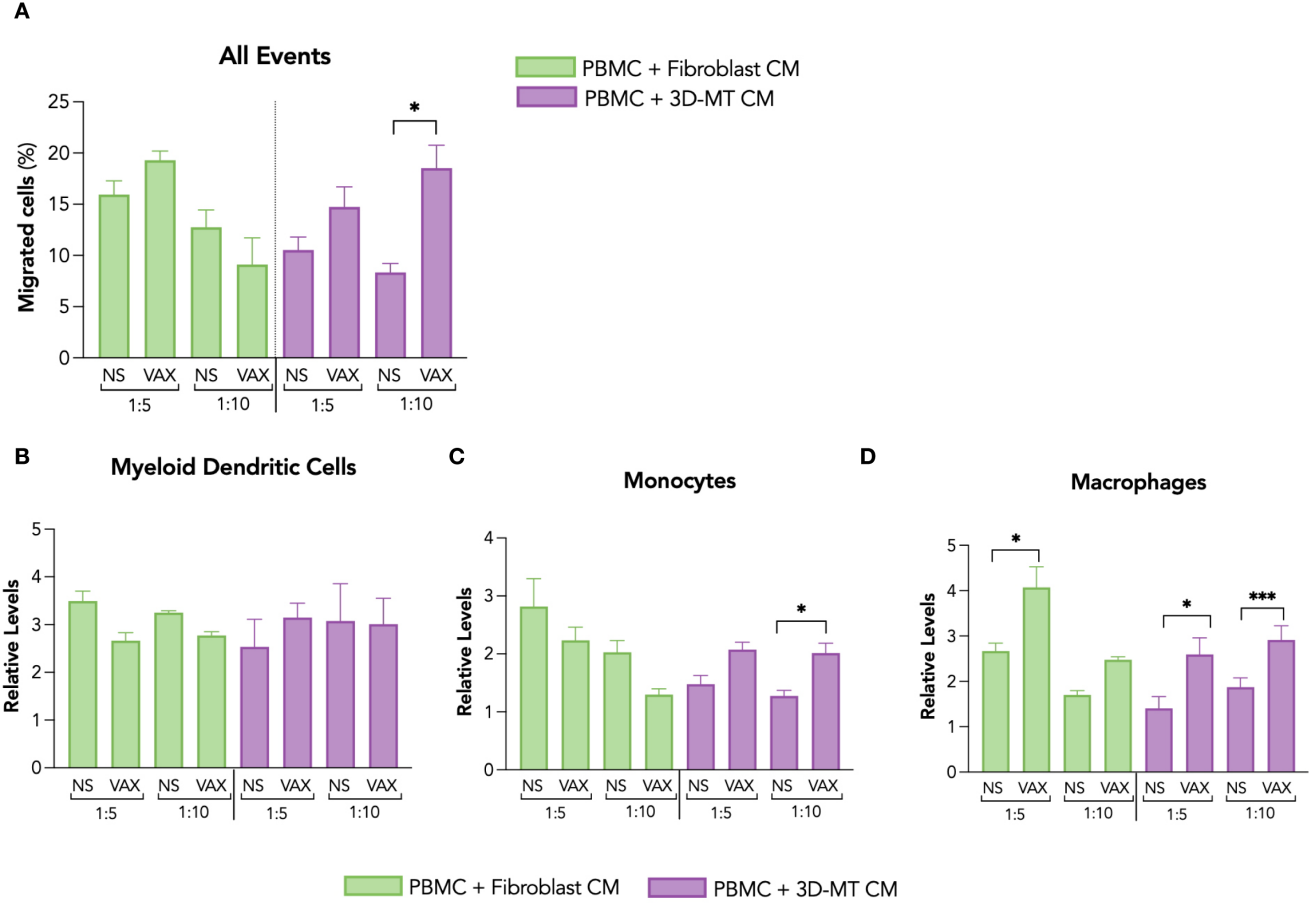
Migration of antigen-presenting cells in response to conditioned media from fibroblasts and 3D muscle-like tissue stimulated with BNT162b2 vaccine. The migration of PBMC was assessed by hemocytometer counting and cytofluorimetric analysis 4 hours (h) post-exposure to conditioned media (CM) collected from fibroblasts and 3D muscle-like tissue (3D-MT) previously stimulated for 24 h with BNT162b2 vaccine (VAX, 1 µg/mL). Values of the migration assay were expressed as percentage of migrated cells with respect to the PBMC input placed in the upper layer of transwells **(A)**. The relative levels of myeloid dendritic cells (mDC) **(B)**, monocytes **(C)**, and macrophages **(D)** were calculated on the total number of cells migrated in the lower layer of the transwell chambers. The results are means ± SEM of three chemotaxis assays (*n* = 3) done with supernatants from three independent experiments. Non-parametric one-way ANOVA with Tukey’s adjustment for multiple comparisons was used to calculate statistical significance differences. The star scale was assigned as follows: *p ≤ 0.05, ***p ≤ 0.001. NS, not stimulated.

To assess whether and how local tissue response shapes the composition of the immune infiltrate, CM from fibroblasts and 3D-MT, either NS or stimulated with the BNT162b2 vaccine, were used in the migration assays (**Figure 4**).

Among the different *in vitro* muscle models, 3D-MT was chosen for collecting CM since its 3D structure more closely resembles the predominant tissue and physiological environment encountered by an intramuscular administered vaccine. Moreover, with this model, several innate immune mediators were found to be rapidly and significantly regulated in response to BNT162b2 vaccine (see **Figure 3**). Thus, PBMC, containing the main immune cells that a vaccine formulation aims to stimulate, were distributed in the upper side of a 5-µm cell culture insert, while in the lower part of the transwell chamber the 1:5 and 1:10 dilutions of CM from fibroblasts and 3D-MT were placed. After 4 h, the total number as well as the type of migrated cells were assessed by hemocytometer counting and cytofluorimetric analysis, respectively. PBMC migration was significantly enhanced by CM from vaccine-injected 3D-MT only at a 1:10 ratio compared to mock-injected 3D-MT (NS) CM, while no increase in the number of migrated PBMC was found in response to fibroblast CM (**Figure 4A**). The cytofluorimetric analysis of migrated cells revealed that CM from both fibroblasts and 3D-MT promoted the migration of APC involved in initiating an Ag-specific adaptive immune response, although at a different extent (**Figures 4B–D**). Irrespective of vaccine-stimulation, soluble factors released under steady-state conditions by fibroblasts and 3D-MT promoted mDC recruitment (**Figure 4B**). On the contrary, CD14^+^ monocytes (**Figure 4C**) and, particularly, CD11b^+^CD14^-^ macrophages (**Figure 4D**) were significantly recruited by CM from both vaccine-exposed fibroblasts (at 1:5 ratio only) and 3D-MT (at both ratio) compared to CM from NS cells. These findings suggest that the combined action of soluble factors released by stroma and tissue compartments impacts on the composition of the immune infiltrate at the injection site, which is characterized by constitutive mDC migration and vaccine-driven recruitment of monocytes and macrophages.

### 3.3 Conditioned media from fibroblasts and 3D muscle-like tissue stimulated with BNT162b2 vaccine differentially regulate antigen-presenting cell immunophenotype

Once recruited at the vaccine injection site, APC can get in contact either with the mediators released by vaccine-stimulated muscle cells or fibroblasts as well as with the vaccine itself present in the extracellular environment.

**Figure 5:**
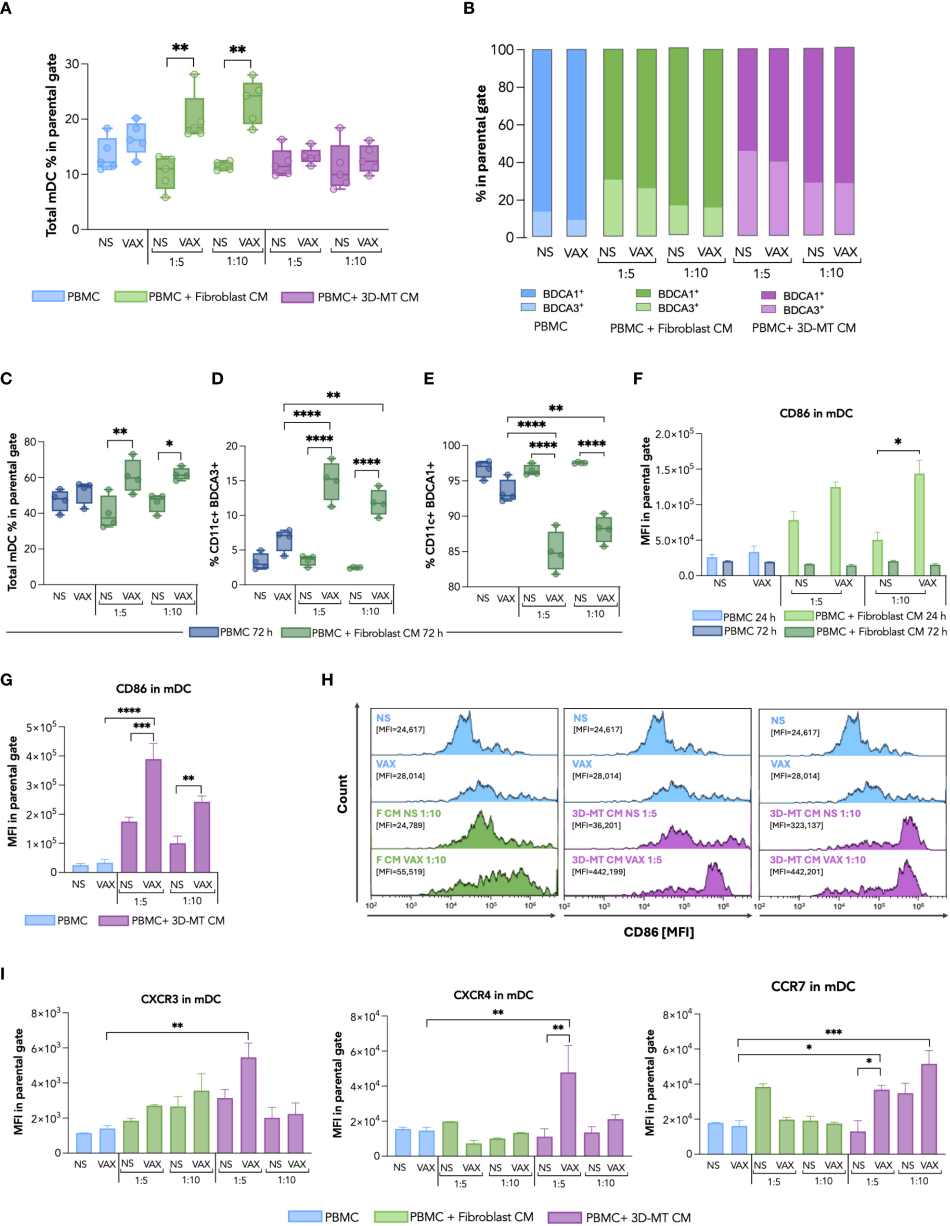
Dendritic cell immunophenotype in response to BNT162b2 vaccine or conditioned media from BNT162b2-vaccine-exposed fibroblasts and 3D muscle-like tissue. Peripheral blood mononuclear cells (PBMC) were left not stimulated (NS) or stimulated for 24 h (h) with BNT162b2 vaccine (VAX, 1 µg/mL) or with conditioned media (CM) at ratios of 1:5 and 1:10, collected from fibroblasts or 3D muscle-like tissue (3D-MT), NS or VAX-treated (1 µg/mL) for 24h. Then, the frequency of total myeloid dendritic cells (mDC) (CD11c^+^ cells) **(A)** and of BDCA1^+^ and BDCA3^+^ subpopulations **(B)** was determined by cytofluorimetric analysis. Similarly, the frequency of total mDC **(C)**, BDCA1^+^ and BDCA3^+^ subpopulations **(D, E)** was analyzed in PBMC NS or treated for 72 h with VAX (1 µg/mL) or with CM from fibroblasts NS or exposed for 72 h to the VAX. For total mDC at 24 h and for mDC subpopulations at 72 h **(A, C–E)**, the results are shown as median values ± interquartile range, while for mDC subsets at 24 h **(B)**, the results shown in the bar chart are mean values of five independent experiments. The mean fluorescence intensity (MFI) of CD86 in total mDC **(F, G)** was expressed as means ± standard error of the mean (SEM) of four independent experiments (*n* = 4). Representative histograms of CD86, out of four experiments separately performed, are shown on selected experimental conditions **(H)**. In particular, the mDC CD86 expression in NS PBMC as well as in PBMC stimulated with VAX was compared with results obtained in PBMC treated with CM from unstimulated (F CM NS) or vaccine-treated (F CM VAX) fibroblasts used at ratio 1:10, while both ratios 1:5 and 1:10 were represented for CM from not stimulated (3D-MT CM NS) or BNT162b2-injected (3D-MT CM VAX) 3D-MT. The MFI of CXCR3, CXCR4, and CCR7 **(I)** in total mDC (CD11c^+^) represented as means ± SEM of three independent experiments (*n* = 3). Non-parametric one-way ANOVA with Tukey’s adjustment for multiple comparisons was used to calculate statistical significance differences. The star scale was assigned as follows: *p ≤ 0.05, **p ≤ 0.01, ***p ≤ 0.001, ****p ≤ 0.001.

**Figure 6:**
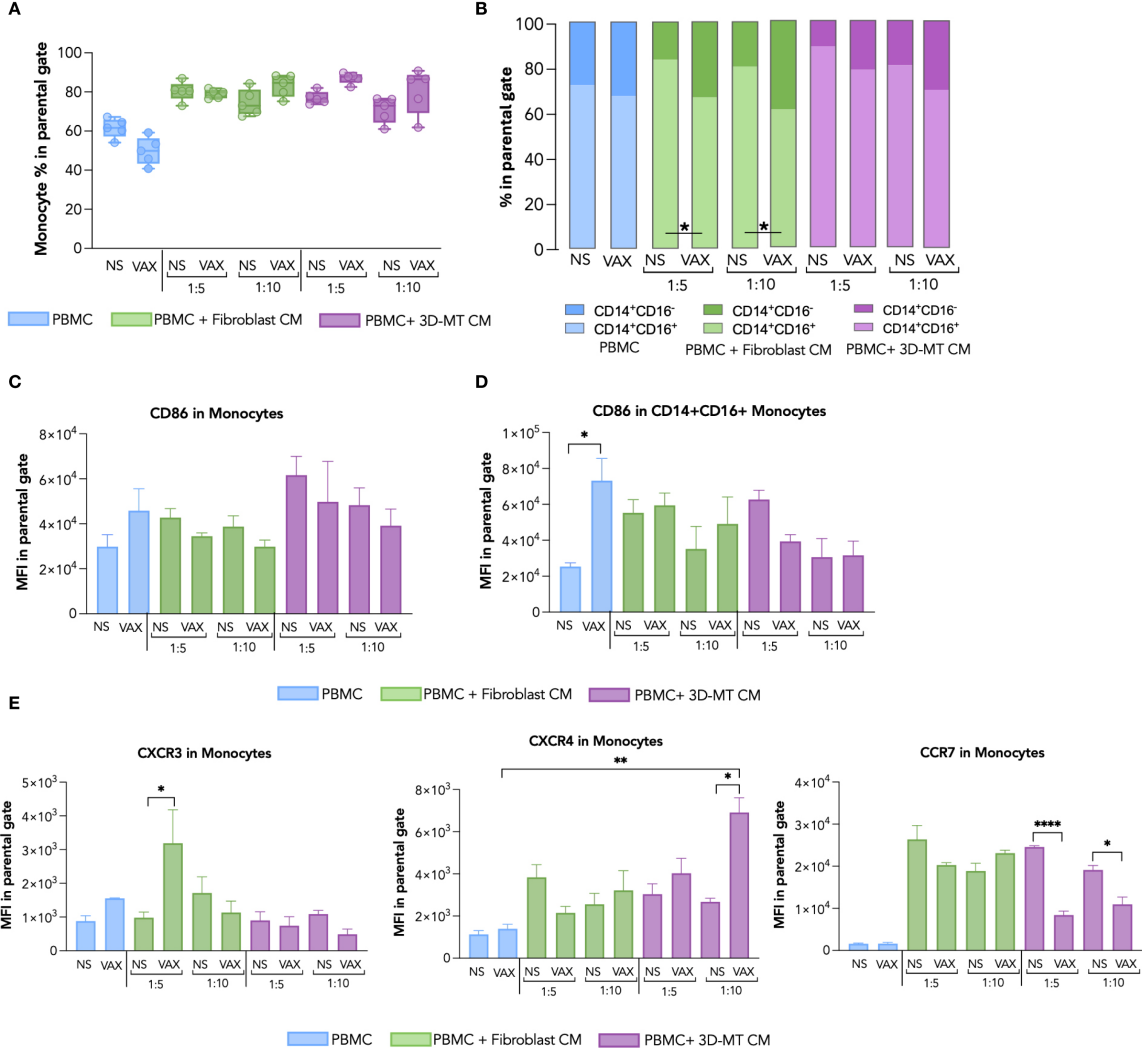
Monocyte immunophenotype in response to BNT162b2 vaccine or conditioned media from BNT162b2-vaccine-treated fibroblasts and 3D muscle-like tissue. Peripheral blood mononuclear cells (PBMC) were left not stimulated (NS) or stimulated for 24 hours (h) with BNT162b2 vaccine (VAX, 1 µg/mL) or, conditioned media (CM), at ratio 1:5 and 1:10, collected from fibroblasts or 3D muscle-like tissue (3D-MT) NS or VAX-treated (1 µg/mL) for 24h. The frequency of total monocytes (CD14^+^ cells) **(A)** and of classical (CD14^+^CD16^-^) and inflammatory (CD14^+^CD16^+^) subpopulations **(B)** was studied by flow cytometric analysis. For total monocytes **(A)**, the results are shown as median values ± interquartile range, while for monocyte subsets **(B)**, the results shown in the bar chart are mean values of five independent experiments (*n* = 5). The mean fluorescence intensity (MFI) of CD86 measured in total CD14^+^ **(C)** and inflammatory CD14^+^CD16^+^ **(D)** was expressed as means ± standard error of the mean (SEM) of four independent experiments (*n* = 4). The MFI of CXCR3, CXCR4, and CCR7 **(E)** was assessed in total monocytes (CD14^+^) and represented as means ± SEM of three independent experiments (*n* = 3). Non-parametric one-way ANOVA with Tukey’s adjustment for multiple comparisons was used to calculate statistical significance differences. The star scale was assigned as follows: *p ≤ 0.05, **p ≤ 0.01, ****p ≤ 0.001.

**Figure 7:**
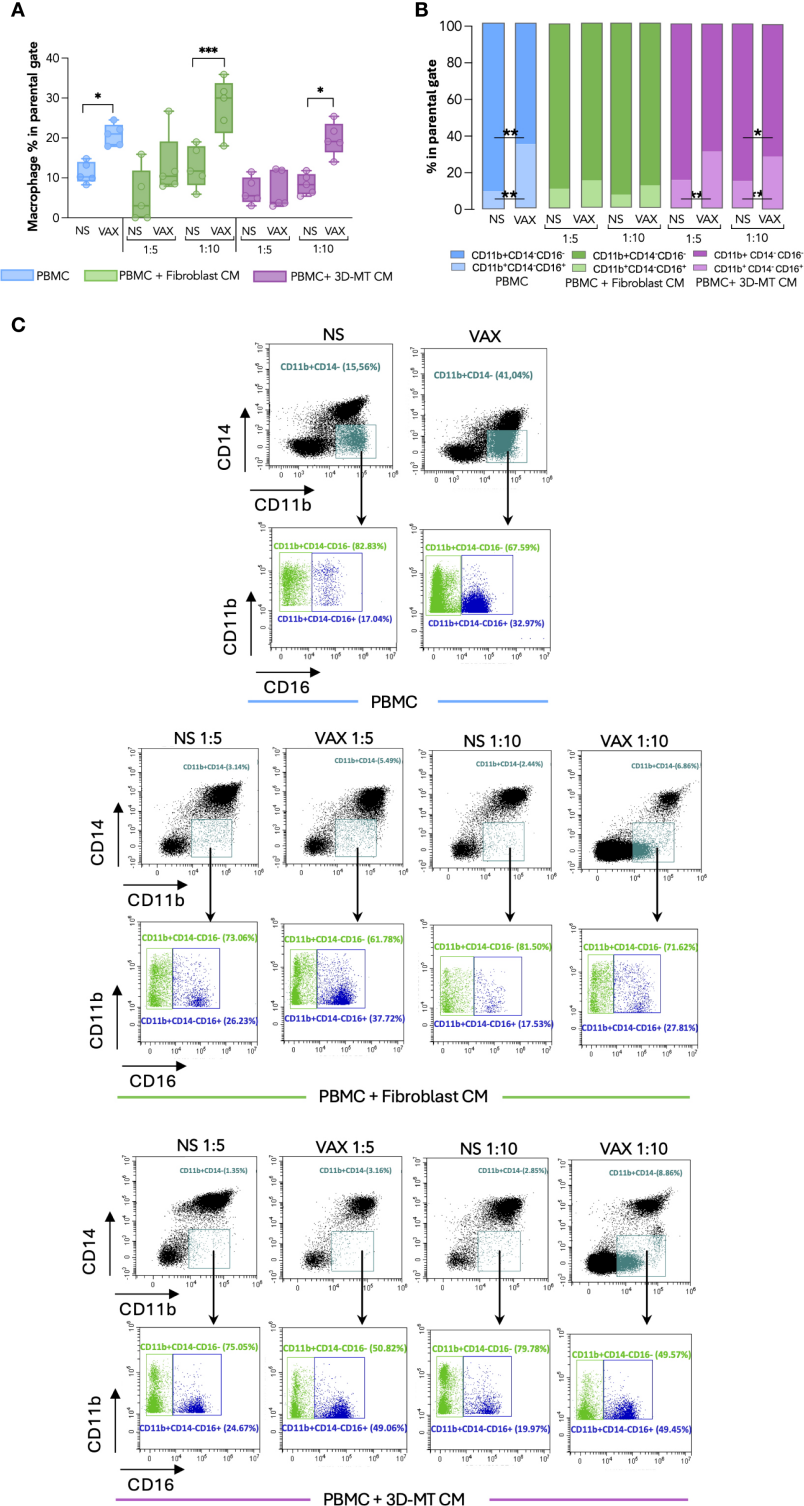
Macrophage immunophenotype in response to BNT162b2 vaccine or conditioned media from BNT162b2-vaccine-treated fibroblasts and 3D muscle-like tissue. Peripheral blood mononuclear cells (PBMC) were left not stimulated (NS) or stimulated for 24 hours (h) with BNT162b2 vaccine (VAX, 1 µg/mL) or conditioned media (CM) at ratios of 1:5 and 1:10 collected from fibroblasts or 3D muscle-like tissue (3D-MT) NS or VAX-treated (1 µg/mL) for 24h. The frequency of total macrophages (CD11b^+^CD14^-^ cells) **(A)** and of classical (CD11b^+^CD14^-^CD16^-^) and activated/inflammatory (CD11b^+^CD14^-^CD16^+^) subpopulations **(B)** was studied by flow cytometric analysis. For total macrophages **(A)**, the results are shown as median values ± interquartile range, while for macrophage subsets **(B)**, the results shown in the bar chart are mean values of five independent experiments (*n* = 5). Non-parametric one-way ANOVA with Tukey’s adjustment for multiple comparisons was used to calculate statistical significance differences. The star scale was assigned as follows: *p ≤ 0.05, **p ≤ 0.01, ***p ≤ 0.001. Representative dot plots of total macrophages and macrophage subsets gated on CD11b^+^CD14^-^ **(C)**. Classical macrophages (CD11b^+^CD14^-^CD16^-^) are indicated in green and the activated/inflammatory macrophages (CD11b^+^CD14^-^CD16^+^) in blue.

Firstly, we conducted a comparative immunophenotypic analysis of APC populations—namely, mDC (**Figure 5**), monocytes (**Figure 6**), and macrophages (**Figure 7**)—in PBMC stimulated for 24 h with BNT162b2 vaccine (1 µg/mL) or with CM from fibroblasts and 3D-MT (1:5 and 1:10 dilutions).

mDC were considered as CD11c^+^ cells in the CD14^-^ gate and further classified in the two main BDCA1^+^ and BDCA3^+^ subsets, known respectively for initiating T cell responses (both CD4^+^ and CD8^+^) and non-self Ag cross-presentation to CD8^+^ T cells (27) (**Figures 5A, B**). In vaccine-treated PBMC, a modest increase in the percentage of total CD11c^+^ mDC was observed (**Figure 5A**), mainly reflecting the variation in BDCA1^+^ subpopulation, the most representative mDC (**Figure 5B**, in light blue). A different picture emerged in PBMC stimulated with CM collected from fibroblast cultures treated for 24 and 72 h with the vaccine. In particular, CM from fibroblasts treated for both 24 and 72 h increased the total CD11c^+^ mDC percentage (**Figures 5A, C**). Although an enhancement in BDCA1^+^ cell frequency appeared upon 24 h of stimulation with CM from 24-h vaccine-treated fibroblasts, the 72-h incubation of PBMC with CM collected from 72 h BNT162b2-vaccine-exposed fibroblasts fostered the enrichment of the BDCA3^+^ mDC subpopulation (**Figure 5B** in light green and **Figure 5D** in green) and reduction of the BDCA1^+^ mDC subset (**Figure 5E**, in green). However, CM from 24-h-treated fibroblasts only (1:10 ratio) drive a vaccine-specific enhancement of the co-stimulatory marker CD86 MFI in total CD11c^+^ mDC, indicating APC activation (**Figures 5F, H**). Although CM from BNT162b2-treated 3D-MT did not modify mDC frequency (**Figures 5A, B**, in purple), a significant enhancement of the CD86 expression was observed in total CD11c^+^ mDC at both 1:5 and 1:10 dilutions (**Figures 5G, H**). It is worth noting that CM from vaccine-stimulated 2D MT did not affect either the mDC frequency (**Supplementary Figures S5A, B**) or the enhancement in CD86 expression on treated PBMC (**Supplementary Figure S5C**).

Considering that CD86 modulation was already evident in mDC after 24 h of stimulation with CM from vaccine-exposed fibroblasts and 3D-MT, we additionally extended our analysis at this time point to include the chemokine receptors CXCR4, CCR7, and CXCR3, which control APC recruitment into peripheral lymph nodes for the former and into inflamed tissues for the latter (**Figure 5I**). While the expression of these chemokine receptors was not markedly modulated in mDC by either direct vaccine exposure or fibroblast-derived CM treatment (**Figure 5I**), a significant enhancement was instead observed after the treatment with CM from vaccine-injected 3D-MT (**Figure 5I**).

In monocytes, the direct BNT162b2 vaccine stimulation leads to a modest reduction of total CD14^+^ cells and of the inflammatory monocyte CD14^+^CD16^+^ subset (**Figures 6A, B**). A significant decrease in CD14^+^CD16^+^ cells coupled with an increase in classical, not inflammatory, CD14^+^CD16^-^ monocytes was observed after the exposure of PBMC to CM from BNT162b2-vaccine-treated fibroblasts or 3D-MT as compared to the control CM (**Figures 6A, B**). No significant variations in the frequency of total CD14^+^ monocytes or in CD14^+^ subsets were triggered by CM from 2D MT (**Supplementary Figures S5D, E** in pink). By evaluating CD86 expression, no modulation was detected in total CD14^+^ (**Figure 6C**), while a significant enhancement was observed in the inflammatory CD14^+^CD16^+^ subset only in response to the BNT162b2 vaccine (**Figure 6D**, **Supplementary Figure S5F**). The expression of the chemokine receptors CXCR3, CXCR4, and CCR7 was instead mainly influenced in CD14^+^ monocytes by CM rather than the direct vaccine treatment (**Figure 6E**). CM from vaccine-treated fibroblasts robustly upregulated CXCR3, while CM from vaccine-injected 3D-MT significantly increased CXCR4 but downregulated the CCR7 expression (**Figure 6E**).

To further dissect *in vitro* the local immune response and assess whether the observed reduction in CD14^+^CD16^+^ monocytes could reflect differentiation into macrophages (28, 29), a cytofluorimetric analysis was conducted and revealed that the percentage of CD14^-^CD11b^+^ macrophages doubled in BNT162b2-stimulated PBMC compared to unstimulated cells (**Figures 7A, C**). Specifically, the frequency of activated CD16^+^ macrophages was significantly enhanced, while the immature CD16^-^ subset percentage was diminished (**Figures 7B, C**). A similar increase in macrophage frequency was observed upon PBMC exposure to CM from BTN162b2-stimulated fibroblasts and 3D-MT at a 1:10 ratio, with the latter inducing a significant reduction of CD16^-^ immature macrophages and the concomitant increase of CD16^+^ activated macrophages (**Figures 7A–C**). Although not statistically significant, fibroblast CM produced a similar trend (**Figures 7B, C**). Conversely, CM from 2D MT failed to modulate in macrophage frequency or subset balance (**Supplementary Figures S5G–I**).

Altogether these results indicate that following vaccine intramuscular administration, factors secreted at the local level by both stromal and tissue-specific cells mainly shape the APC activation profile, while the direct interaction of immune cells with BNT162b2 vaccine mostly drives the differentiation of monocytes into activated macrophages.

### 3.4 Conditioned media from BNT162b2-stimulated fibroblasts or 3D muscle-like tissue induced in PBMC an innate module related to protective immune responses

**Figure 8:**
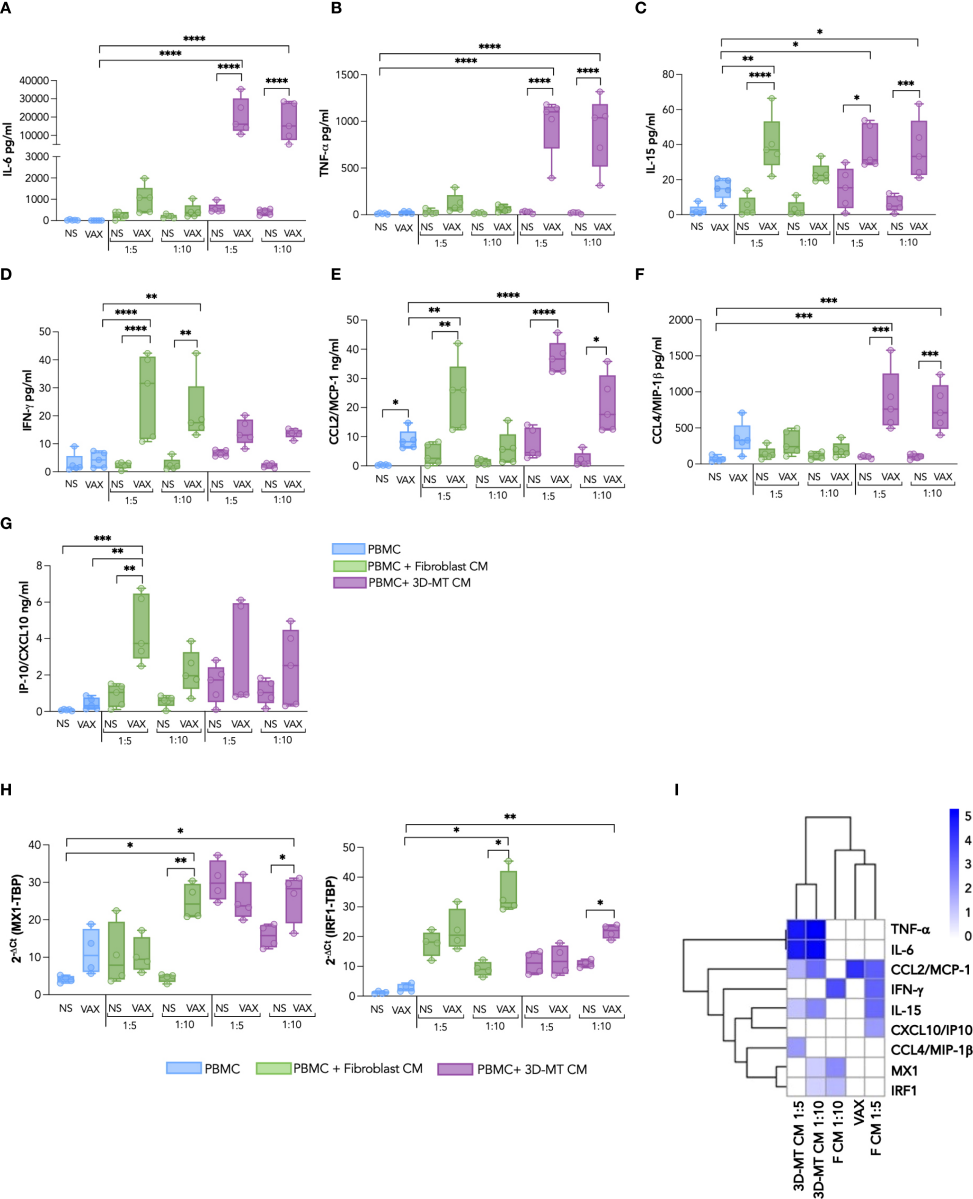
Panel of cytokines and chemokines produced by PBMC after exposure to BNT162b2 vaccine or conditioned media from BNT162b2-vaccine-treated fibroblasts and 3D muscle-like tissue. Peripheral blood mononuclear cells (PBMC) were left not stimulated (NS) or stimulated for 24 h **(H)** with BNT162b2 vaccine (VAX, 1 µg/mL) or with conditioned media (CM) from fibroblasts or 3D muscle-like tissue (3D-MT) respectively unstimulated (NS) or treated with VAX (1 µg/mL) for 24h All CMs were used at ratio 1:5 and ratio 1:10. The levels of cytokines IL-6 **(A)**, TNF-α **(B)**, IL-15 **(C)**, and IFN-γ **(D)** and of chemokines CCL2/MCP-1 **(E)**, CCL4/MIP-1β **(F)**, and CXCL10/IP-10 **(G)** were measured in culture supernatants. MX1 and IRF1 **(H)** expression was determined at 24 h by quantitative RT-PCR. The results are shown as median values ± interquartile range of five experiments (*n* = 5) separately performed with fibroblasts and 3D-MT CM from three independent experiments. Non-parametric one-way ANOVA with Tukey’s adjustment for multiple comparisons was used to calculate statistical significance differences. The star scale was assigned as follows: *p ≤ 0.05, **p ≤ 0.01, ***p ≤ 0.001, ****p ≤ 0.0001. Heat map representing the log2 fold change of expression of TNF-α, IL-6, IFN-γ, IL-15, CCL2/MCP-1, CXCL10/IP-10, CCL4/MIP-1β, MX1, and IRF1 as compared to the corresponding negative controls according to the color scheme where 0 value (white) corresponds to no variation, while 5 (royal blue) stands for highly modulated and significant markers **(I)**. Dendrograms connect analytes that are significantly regulated with a similar pattern.

Having found that the BNT162b2 vaccine, either directly or through bystander effects mediated by tissue-resident cells, influences the immune cell phenotype and differentiation status, we extended our analysis to the PBMC secretome that could, in turn, act at a local or systemic level to orchestrate the protective immune response. In particular, we focused on the regulation of an early innate immune module previously shown to predict protective humoral responses to the BNT162b2 vaccine *in vivo* (25). To this aim, PBMC were *in vitro* stimulated with the anti-COVID-19 mRNA vaccine or with CM derived from vaccine-treated fibroblasts and 3D-MT (**Figure 8**). Since thawed CM were used in our experimental setup, to evaluate the contribution of the residual BNT162b2 vaccine likely present in the supernatants from vaccine-treated fibroblasts and 3D-MT, PBMC were also stimulated with reconstituted vaccine subjected to a freeze–thaw cycle (FRZ. VAX) at 1 µg/mL (the dose used in the study) and 0.2 and 0.1 µg/mL (corresponding to the estimated vaccine content in CM at ratios of 1:5 and 1:10, respectively) (**Supplementary Figure S6**).

The multiplex analysis of soluble factors contained in the aforementioned innate module, namely, IL-6, TNF-α, IL-15, IFN-γ, MIP-1β, MCP-1, and IP-10, indicated that stimulation of PBMC with CM from fibroblasts and 3D-MT resulted in a consistent modulation of PBMC secretome. In contrast, direct stimulation with BNT162b2 mRNA vaccine had no or limited effect except for the monocyte chemoattractant MCP-1 induction (**Figures 8A–G**). Upon treatment with CM from vaccine-injected 3D-MT, PBMC displayed a robust enhancement in the release of the pro-inflammatory cytokines IL-6 and TNF-α (**Figures 8A, B**) as well as the macrophage/monocyte chemoattractant MIP-1β (**Figure 8F**). CM from vaccine-treated fibroblasts and 3D-MT also significantly potentiated the secretion of IL-15, a pleiotropic cytokine involved in inflammation and in priming/maintenance of adaptive immunity (**Figure 8C**) and of MCP-1 (**Figure 8E**). Interestingly, CM from BNT162b2-vaccine-treated fibroblasts significantly and specifically promoted in PBMC the co-secretion of the type II IFN-γ and of the IFN-inducible chemokine IP-10 (**Figures 8D, G**). Consistent with these findings and with the observed expression of type I IFNs (IFN-αs and IFNB1) (**Supplementary Figure S4**), we also found a significant modulation of IFN-inducible genes MX1 and IRF1. These genes are part of the innate immune panel previously identified as predictive of BNT162b2-induced protection *in vivo* (25) and were significantly upregulated upon PBMC exposure to CM from vaccine-stimulated fibroblasts (1:10 ratio) and vaccine-injected 3D-MT (**Figure 8H**). In contrast, FRZ. VAX had no significant effect on any of the molecules analyzed in **Figure 8**, supporting the central role of local tissue responses in shaping peripheral immune cell secretome (**Supplementary Figure S6**).

Finally, to explore the inter-relationship between direct and indirect BNT162b2-vaccine-induced effects on the early innate immune module in PBMC, we constructed a heat map using the Log2(fold change) of factors with FDR <0.01 (**Figure 8I**). Hierarchical clustering additionally highlights that (I) BNT162b2 vaccine alone induces MCP-1 chemokine, (II) tissue-resident muscle cells prime PBMC to generate an immunostimulatory soluble milieu including the innate cytokines TNF-α, IL-6, and IL-15 as well as the chemotactic factor MIP-1β, and (III) stromal compartment, here recapitulated by human primary fibroblasts, further enhances PBMC responses by promoting the expression of IFN-γ and other innate immune mediators belonging to the IFN signature, such as IP-10, MX1, and IRF1. Collectively, these findings indicate that interaction among immune, stromal, and tissue compartments can recapitulate *in vitro* the early serum/blood signature associated with vaccine-induced protection observed in BNT162b2 recipients *in vivo*.

## 4 Discussion

Molecular networks belonging to innate immunity and correlating with later adaptive vaccine-specific protective humoral and cellular responses have been identified by system vaccinology, leveraging the integration of multi-omics analysis with computational approaches (30–34). The first studies focused on subjects that get vaccinated with seasonal influenza and yellow fever vaccines, but in recent years, this field rapidly expanded, including individuals immunized with diverse vaccine platforms targeting both viral and bacterial pathogens (33).

Considering that blood modules identified by system vaccinology are rapidly induced in systemic circulation following vaccine administration, a contribution of cells encountering vaccine at the administration site—i.e., stroma and tissue-resident cells—in the modulation of this signature might be envisaged. Indeed *in vivo* vaccine response results from multiple environmental interactions among different cell types and extracellular matrix. Therefore, *in vitro* models designed for studying vaccine responses should provide a sufficient degree of biomimicry to emulate the fine-tuned and highly interdependent immunoregulation of the human body (35).

**Figure 9:**
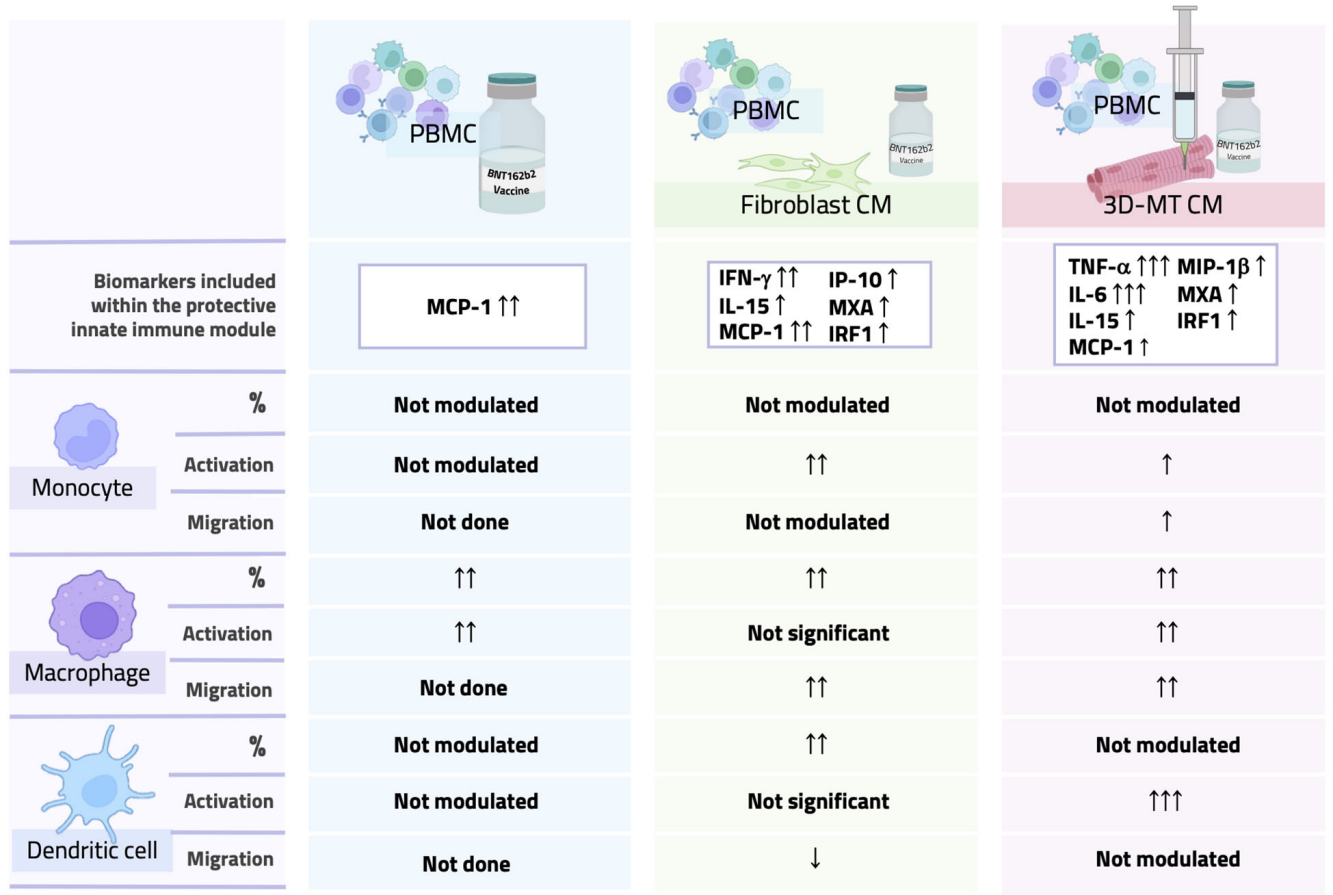
Overall comparison of PBMC phenotype after direct stimulation with BNT162b2 vaccine or with conditioned media from vaccine-treated fibroblasts and 3D muscle-like tissue. Graphical table summarizing data obtained by direct stimulation of PBMC with the BNT162b2 vaccine or with conditioned media (CM) from vaccine-treated fibroblasts or 3D muscle-like tissue (3D-MT). The table contains a schematic representation of results for a panel of biomarkers linked to a protective innate immune module and monocyte, macrophage, and dendritic cell migratory capacity and activation/maturation profile.

In the attempt to evaluate this non-immune cell contribution in the shaping of early immune response to BNT162b2 vaccine, here we interrogated human-cell-based immune-relevant *in vitro* models composed of vaccine-exposed PBMC, human primary fibroblasts and 3D-MT. By these heterologous cell-based systems, we simulated *in vitro* the intramuscular administration of BNT162b2 mRNA vaccine to validate the expression of the early innate immune module, featuring IFN-inducible genes MX1 and IRF1, the serum cytokines IL-6, TNF-α, IL-15, and IFN-γ and the chemokines IP-10, MIP-1β, and MCP-1 (**Figure 9**) that we and others have previously found in the sera and blood of vaccinees associated with vaccine-induced protective Ab titers (25, 36, 37).

An intriguing aspect emerging from our study is that the early immune signature associated with BNT162b2-vaccine-driven protection not only results from vaccine direct sensing by immune cells but also requires the crosstalk among immune compartment, stroma, and tissue-resident cells, here represented by fibroblasts and the 3D-MT model respectively, thus highlighting that many factors contribute to vaccine immunogenicity.

The concept that immune functions are not unique features of hematopoietic cells is now well accepted and has been systematically investigated in an organ-specific study that observed a massive regulation of immune-related genes at both steady-state level and in response to stimuli with structural cells as key regulators of organ-specific immune responses (38). In the case of muscle tissue, the active cross-talk among myogenic, vascular, immune, and fibrotic cells involving paracrine/autocrine action of myokines and anti-inflammatory signals as well as contact interactions has been described and modeled, particularly along muscle injury repair (15, 39, 40).

The large-scale administration of BNT162b2 vaccine to face the COVID-19 pandemic has provided an exceptional opportunity to deepen our understanding on mRNA-based vaccine mechanism of action. Regarding the expression of SARS-CoV-2 S protein, the BNT162b2-encoded Ag, a biodistribution analysis on vaccinees have reported the presence of S protein in plasma within the first 5 days from immunization and in ipsilateral axillary lymph nodes 2 weeks after vaccine administration (41). Studies conducted on different animal models have revealed that S protein is rapidly and mainly expressed at the injection site and in the liver, spleen, and lungs (41). In this context, our *in vitro* study, providing a snapshot of the events occurring in the first 24 h after vaccine exposure, suggests that S expression at the injection site occurs mainly in fibroblasts. This observation is compatible with the detection, at the injection site, of green fluorescent protein encoded by an mRNA-LNP-based formulation in connective tissue and not in muscle fibers of rodents (42). Similarly, in mRNA-LNP-injected BALB/c mice, SARS-CoV-2 S mRNA was found to be rapidly enriched in stromal cells, particularly in fibroblasts where, differently from other cells, the enrichment lasts up to 16 h post-administration (43). However, even if muscle cells and recruited immune cells do not express S Ag, they sense the mRNA molecules and induce both endosomal and cytoplasmic RNA sensor expression.

It is worth noting that all of the modulations observed in this study refer to the final formulation of the vaccine. Therefore, we cannot distinguish between the effects mediated by the Ag (mRNA) and those mediated by the LNP carrier. However, the possibility that the LNP component by itself contributes to the immune response elicited by the BNT162b2 vaccine cannot be excluded. A recent study conducted in BALB/c mice showed that indeed the ionizable lipid components used in the Moderna (SM-102) and in the Pfizer-BioNTech (ALC-0315) vaccines not only function as delivery systems but also exhibit adjuvant properties (44). Accordingly, in BALB/c mice, it has been proven that the LNP component of mRNA vaccines is responsible for the increased expression of chemokine genes and for the differentiation of two inflammatory fibroblast populations at the intramuscular injection site (43).

A number of studies involving non-human primate and mice models revealed that neutrophils, classic monocytes, macrophages, and mDC are the major immune cells infiltrating the tissues upon mRNA-LNP-based vaccine administration (8, 45, 46). In line with these observations, the overall milieu—most likely containing MCP-1/CCL2 and IP10/CXCL10—conditioned by vaccine-exposed fibroblasts and 3D-MT may recruit within the first 24 h CD14^+^ monocytes and particularly CD14^-^CD11b^+^ macrophages. In addition, PBMC direct exposure to BNT162b2 vaccine or to soluble factors released from vaccine-conditioned stromal and tissue compartments led to the differentiation of CD14^-^CD11b^+^CD16^+^ mature macrophages. Factors secreted at the local level also contribute to the upregulation of CXCR3 and the concomitant reduction of CCR7 expression in monocytes, thus acquiring a phenotype generally associated with infiltration into the inflamed tissue and, in turn, with their local differentiation into macrophages (47, 48). Regarding mDC phenotype, no effects were elicited by the BNT162b2 vaccine by itself, while factors locally released by fibroblasts and 3D-MT drive DC differentiation and the increase of the co-stimulatory marker CD86 and chemokine receptors, respectively, thus contributing to DC activation and acquisition of migratory capacity. In this context, the increased secretion of RANTES observed in BNT162b2-vaccine-treated muscle compartment well correlates with the upregulation of CXCR3 chemokine receptor on the DC surface. Moreover, muscle-cell-secreted factors also foster CCR7 expression on DC, thus indicating the acquisition of migratory capacity to lymphoid organs.

Concerning the role of stroma compartment, fibroblasts are increasingly recognized as active players influencing broader immune organization and functionality at the tissue level where, through the acquisition of stromal organizer-like features, they support the establishment of local immune niches and promote Ag presentations in different contexts (18, 49–52). Here the BNT162b2-induced expression of MX1 in fibroblasts coupled with the robust increase of total CD11c^+^ mDC at 24 and 72 h after PBMC exposure to CM from vaccine-treated fibroblasts is in line with the transcriptome analysis at a single-cell level conducted by Kim and collaborators (43). These data revealed a rapid induction of IFNB1 in the fibroblasts of vaccine-injected mice, approximately accounting for 50% of the IFNB1-expressing cells at the local level after an intramuscular administration of the mRNA vaccine (43). Interestingly, type I IFN, locally produced, was linked to the specific induction of a mDC population expressing type I IFN-inducible genes at the injection site as well as in draining lymph nodes (43). In addition, our immunophenotypical analysis also indicated that factors released by fibroblasts in response to the vaccine at a longer exposure time (namely 72 h) increase the frequency of BDCA3+ mDC subpopulation, deputed to cross-present non-self-Ag to CD8^+^ T cells (27). In the combined *in vitro* systems where the communication between vaccine-injected 3D-MT and PBMC and vaccine-treated fibroblasts and PBMC occurred, a significant increment in IFN-inducible genes was detected. As indicated by the hierarchical clustering analysis, soluble factors, released in the first 24 h following BNT162b2 vaccine exposure by fibroblasts, rather than 3D-MT, seems to promote in PBMC an IFN-stimulated signature, here recapitulated by the IFN-inducible genes MX1 and IRF1, the chemokine IP10, the cytokine IL-15, and the type II IFN, IFN-γ itself. On the other hand, the interaction of muscle compartment with BNT162b2 vaccine contributes to the establishment of an inflammatory and immunostimulatory soluble milieu likely influencing monocyte and, especially, macrophage phenotype via the significant enhancement of the pro-inflammatory cytokines TNF-α and IL-6 and the chemotactic factors MCP-1 and MIP-1β (53, 54). In this context, it has been reported that MIP-1β can synergize with RANTES and IFN-γ to ultimately mount Ag-specific CD8^+^ T cell responses via the involvement of NK cells (54).

We acknowledge that the *in vitro* systems used in this study represent a simplified model of human physiology and only partially contribute to the limited availability of multicellular *in vitro* models addressing the complexity of human immune responses to vaccination. This limitation is largely due to the restricted accessibility to MP and autologous stromal and blood cells as well as to the difficulty in replicating the contribution of resident immune cells in the early phases of vaccine-induced immune response. However, this study constitutes, to our knowledge, the first evidence of human primary-cell-based *in vitro* models emulating BNT162b2 vaccine intramuscular administration and evaluating whether and how local responses can (I) shape the composition of the immune infiltrate at the injection site and (II) influence the systemic immune signature in the early phases soon after vaccine administration. Our study highlights the pivotal role of non-immune cells residing at the injection site in influencing the immune response to mRNA vaccination. This observation suggests that, in order to enhance the immunogenicity of next-generation mRNA-based therapeutics, it may be crucial to consider and target not only immune cells but also stromal and tissue-resident components as integral players in shaping vaccine efficacy. These data may also provide evidence for the exploitation of these *in vitro* models, alone or in combination with animal studies, in different phases of the vaccine timeline. This would be of particular interest especially when animal models fail to provide reliable correlates of protection given the physiological inter-species differences in immune responses (34, 55, 56).

Further improvements of our proposed models will be supported by the rapid technological advancements in 3D-culture technologies, tissue engineering, and microfluidic systems that will provide the necessary tools to develop a 3D-MT composed of both tissue and stromal cells, thus overcoming an inherent limitation of our study concerning the evaluation of stromal compartment contribution in a 2D format only. The integration of these technological innovations with data-driven and mechanistic computational modeling approaches will offer the possibility to re-create integrated *in vitro*/*in silico* experimental environments. These advanced systems will more accurately reproduce the structural and functional dynamics of early vaccine-driven immune modulations associated with protective responses.

## Data Availability

The raw data supporting the conclusions of this article will be made available by the authors, without undue reservation.
