# Supplementary material for: A biomimetic model composed of injectable 3D muscle-like tissue, stromal and immune cells for recapitulating the rapid immune signature predictive of mRNA vaccine immunogenicity
Source: Front Immunol. 2025 Oct 10;16:1651095. doi: 10.3389/fimmu.2025.1651095 (PMC12572801; doi:10.3389/fimmu.2025.1651095)
Supplement: Supplementary file 1 [file DataSheet1.pdf]

1 Supplementary Figures

1.1 Supplementary Figure 1

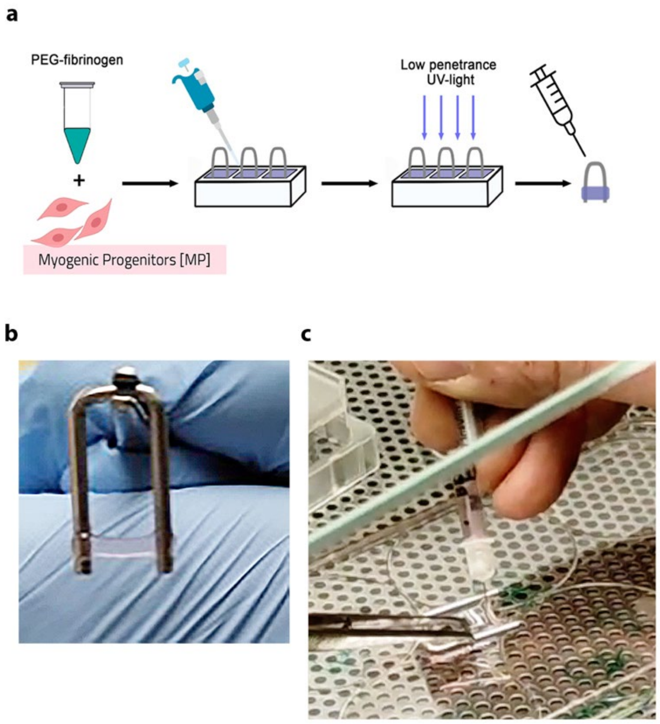

Supplementary Figure 1. BNT162b2 vaccine injection into the 3D muscle-like tissue. Graphical representation of 3D muscle-like tissue (3D-MT) preparation (**a**). In brief, $1x10^6$/ml myogenic progenitors (MP) were combined with 8 mg/ml polyethylene glycol fibrinogen (PF) biomimetic matrix and placed into a Teflon mold containing U-shaped steel support to create a muscle-like structure. The constructs were then left to differentiate for 21 days in cell growth medium (**b**). Subsequently, BNT162b2 vaccine was administered to the 3D-MT constructs by injection with an insulin syringe (31G needle) (**c**).

1.2 Supplementary Figure 2

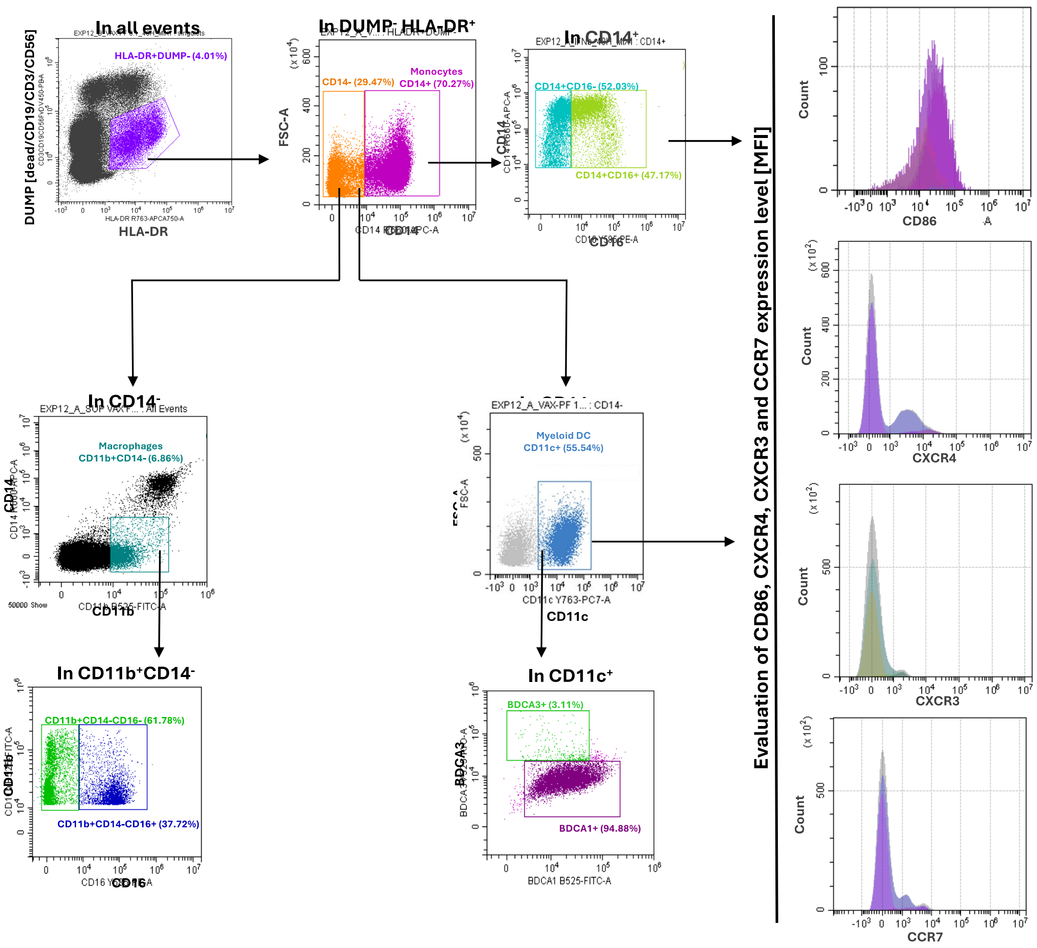

Supplementary Figure 2. Gating strategy employed in cytofluorimetric FACS analysis. Gating strategy employed for studying by flow cytometry monocyte ($CD14^+$), macrophage ($CD11b^+$ $CD14^-$) and myeloid dendritic cell ($CD11c^+$) subsets and activation status.

1.3 Supplementary Figure 3

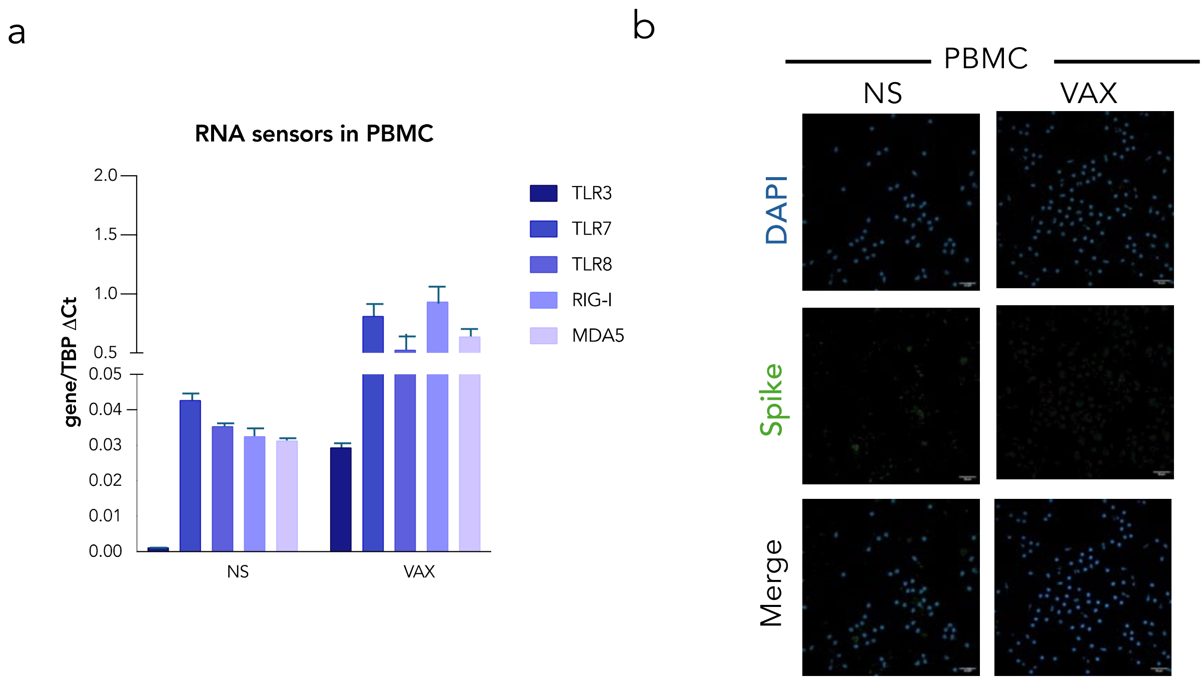

Supplementary Figure 3. Analysis of RNA sensors and SARS-CoV-2 spike protein expression in PBMC stimulated with BNT162b2 vaccine. PBMC were not stimulated (NS) or stimulated with BNT162b2 vaccine (VAX, 1 µg/ml) for 24 hours (h). The expression of the RNA sensors, namely TLR3, TLR7, TLR8, RIG-I and MDA5 was analyzed by quantitative RT-PCR (**a**). Results are means ± standard error of the mean of three experiments separately performed. Immunofluorescence analysis was conducted by confocal microscopy to assess the expression of SARS-CoV-2 spike protein (**b**). PBMC were stained with DAPI (blue) to identify nuclei and with an anti-spike antibody (green). Representative images out of three experiments separately performed is shown.

1.4 Supplementary Figure 4

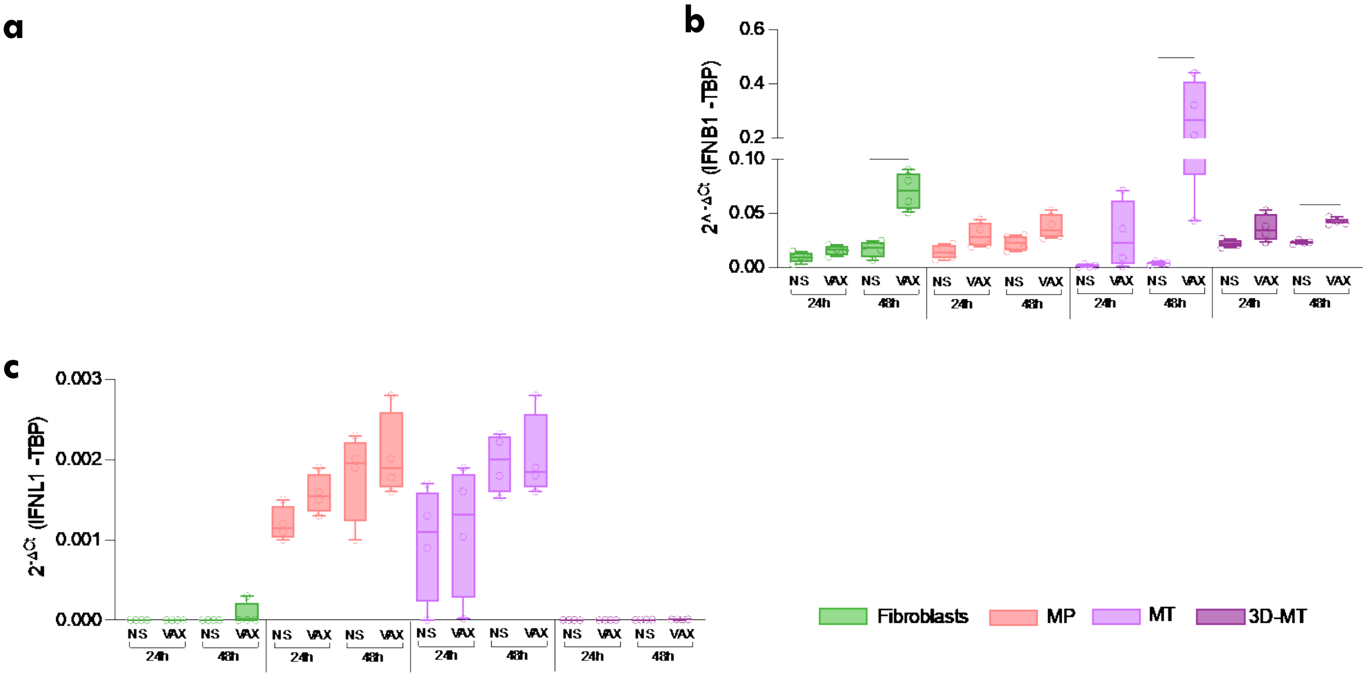

Supplementary Figure 4. Expression of type I and type III IFNs upon stimulation of fibroblast and muscle models with BNT162b2 vaccine. Fibroblasts, myogenic progenitors (MP), myotubes (MT) and 3D-muscle like tissue (3D-MT) were not stimulated (NS) or treated with BNT162b2 vaccine (VAX, 1 µg/ml) for 24 h and 48 h. The production of Interferon (IFN)-αs (**a**) was measured in culture supernatants by ELISA while, the expression of IFNB1 (**b**) and IFNL1 (**c**) was determined by quantitative RT-PCR. Results are shown as median values ± Interquartile range of four independent experiments. Non-parametric One-way ANOVA with Tukey's adjustment for multiple comparisons was used to calculate statistical significance of difference. Star scale was assigned as follow * $p \leq 0.05$, **** $p \leq 0.001$.

1.5 Supplementary Figure 5

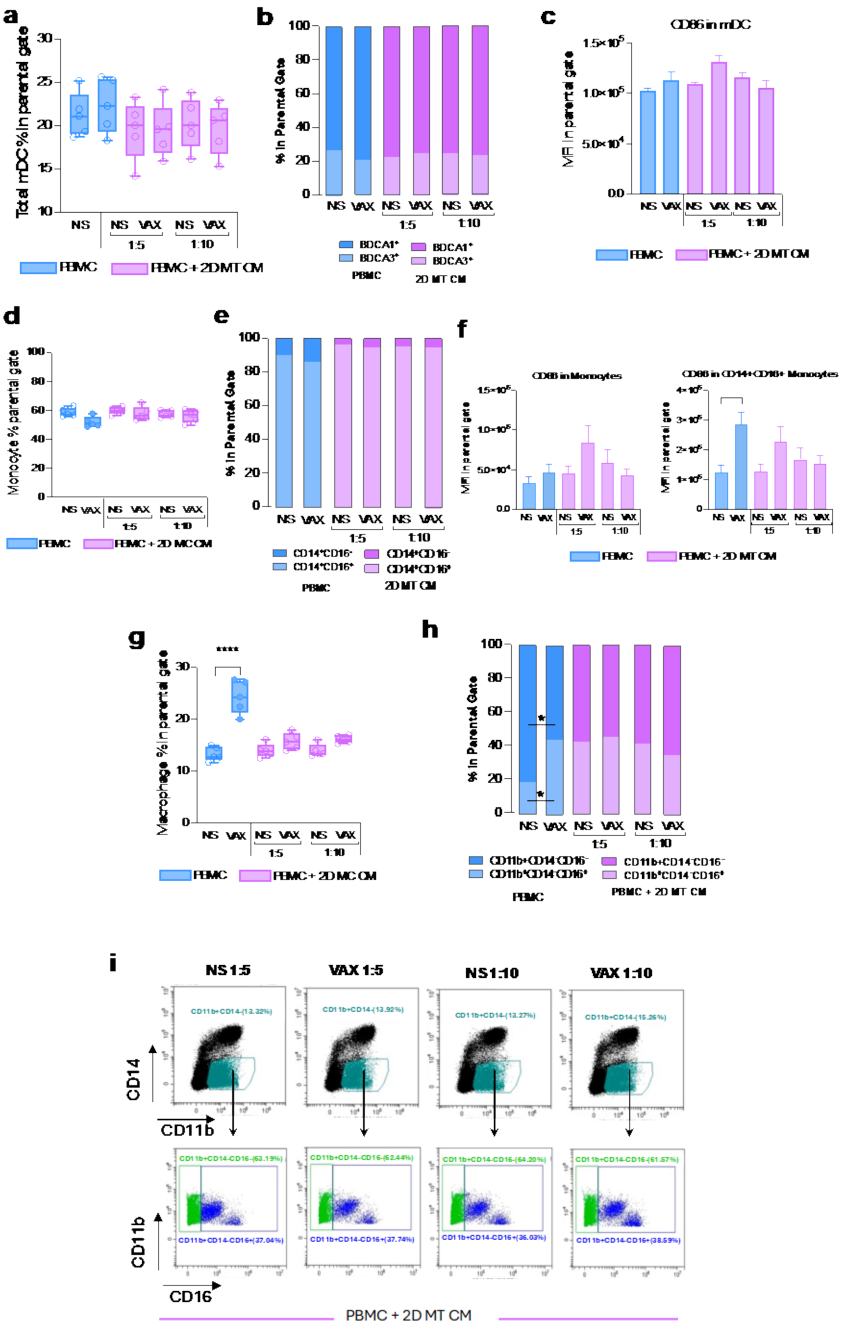

Supplementary Figure 5. Dendritic cell, monocyte and macrophage immunophenotype following stimulation with BNT162b2 vaccine or conditioned media from BNT162b2 vaccine exposed 2D myotubes. Peripheral blood mononuclear cells (PBMC) were left not-stimulated (NS) or stimulated for 24 hours (h) with BNT162b2 vaccine (VAX, 1 µg/ml) or with conditioned media (CM), at ratio 1:5 and 1:10, collected from 2D myotubes (MT) NS or treated with VAX (1 µg/ml) for 24 h. Then, the frequency of total myeloid dendritic cells (mDC) ($CD11c^{+}$ cells) (**a**) and of $BDCA1^{+}$ and $BDCA3^{+}$ subpopulations (**b**), total monocytes ($CD14^{+}$ cells) (**d**) and of classical ($CD14^{+}CD16^{-}$) and

inflammatory ($CD14^+CD16^+$) subpopulations (**e**), total macrophages ($CD11b^+CD14^-$ cells) (**g**) and of classical ($CD11b^+CD14^-CD16^-$) and activated/inflammatory (CD11b+$CD14^-CD16^+$) subpopulations (**h**) was determined by flow cytometric analysis. For total mDC (**a**), monocytes (**d**) and macrophages (**g**) results are shown as median values ± Interquartile range (IQR) while, for mDC, monocyte and macrophage subsets (**b, e, h**), results shown in the bar chart are mean values of five independent experiments. Mean fluorescence intensity (MFI) of CD86 in total mDC (**c**), total (**b**) and inflammatory $CD14^+CD16^+$ (**d**) monocytes was expressed as means ± SEM of four independent experiments. Star scale was assigned as follow * $p \leq 0.05$, **** $p \leq 0.001$. Representative dot-plots of total macrophages and macrophage subsets gated on $CD11b^+$ $CD14^-$ (**c**). Classical macrophages ($CD11b^+CD14^-CD16^-$) are indicated in green, activated/inflammatory macrophages ($CD11b^+CD14^-CD16^+$) in blue.

1.6 Supplementary Figure 6

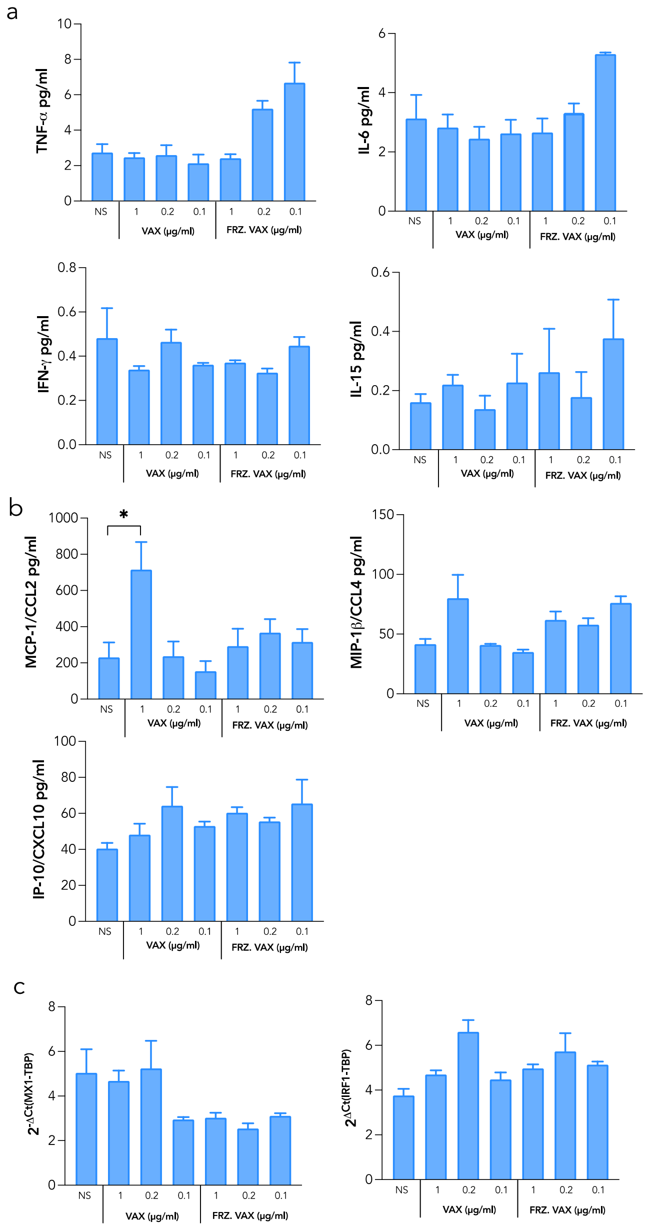

Supplementary Figure 6. Profiling of cytokines and chemokines related to innate immune module in PBMC treated with freshly reconstituted or frozen BNT162b2 vaccine. Peripheral blood mononuclear cells (PBMC) were not stimulated (NS) or stimulated for 24 hours (h) with 1, 0.2 or 0.1 µg/ml of freshly reconstituted (VAX) or frozen BNT162b2 vaccine (FRZ. VAX). Levels of cytokines TNF-α, IL-6, IFN-γ, IL-15 (**a**) and of chemokines CCL2/MCP-1, CCL4/MIP-1β, CXCL10/IP-10, (**b**) were measured in culture supernatants. Mx1 and IRF1 (**d**) expression was determined at 24 h by quantitative RT-PCR. Results are means ± SEM of three experiments separately performed. Non-parametric One-way ANOVA with Tukey's adjustment for multiple comparisons was used to calculate statistical significance differences. Star scale was assigned as follow * $p \leq 0.05$.
